# Point process analysis of noise in early invertebrate vision

**DOI:** 10.1371/journal.pcbi.1005687

**Published:** 2017-10-27

**Authors:** Kris V. Parag, Glenn Vinnicombe

**Affiliations:** Control Group, Department of Engineering, University of Cambridge, United Kingdom; Technion, ISRAEL

## Abstract

Noise is a prevalent and sometimes even dominant aspect of many biological processes. While many natural systems have adapted to attenuate or even usefully integrate noise, the variability it introduces often still delimits the achievable precision across biological functions. This is particularly so for visual phototransduction, the process responsible for converting photons of light into usable electrical signals (quantum bumps). Here, randomness of both the photon inputs (regarded as extrinsic noise) and the conversion process (intrinsic noise) are seen as two distinct, independent and significant limitations on visual reliability. Past research has attempted to quantify the relative effects of these noise sources by using approximate methods that do not fully account for the discrete, point process and time ordered nature of the problem. As a result the conclusions drawn from these different approaches have led to inconsistent expositions of phototransduction noise performance.

This paper provides a fresh and complete analysis of the relative impact of intrinsic and extrinsic noise in invertebrate phototransduction using minimum mean squared error reconstruction techniques based on Bayesian point process (Snyder) filters. An integrate-fire based algorithm is developed to reliably estimate photon times from quantum bumps and Snyder filters are then used to causally estimate random light intensities both at the front and back end of the phototransduction cascade. Comparison of these estimates reveals that the dominant noise source transitions from extrinsic to intrinsic as light intensity increases. By extending the filtering techniques to account for delays, it is further found that among the intrinsic noise components, which include bump latency (mean delay and jitter) and shape (amplitude and width) variance, it is the mean delay that is critical to noise performance. As the timeliness of visual information is important for real-time action, this delay could potentially limit the speed at which invertebrates can respond to stimuli. Consequently, if one wants to increase visual fidelity, reducing the photoconversion lag is much more important than improving the regularity of the electrical signal.

## Author summary

The invertebrate phototransduction system captures and converts environmental light inputs into electrical signals for use in later visual processing. Consequently, one would expect it to be optimised in some way to ensure that only a minimal amount of environmental information is lost during conversion. Confirming this requires an understanding and quantification of the performance limiting noise sources. Photons, which are inherently random and discrete, introduce extrinsic noise. The phototransduction cascade, which converts photons into electrical bumps possessing non-deterministic shapes and latencies, contributes intrinsic noise. Previous work on characterising the relative impact of all these sources did not account for the discrete, causal, point process nature of the problem and thus results were often inconclusive. Here we use non-linear Poisson process filtering to show that photon noise is dominant at low light intensity and cascade noise limiting at high intensity. Further, our analysis reveals that mean bump delay is the most deleterious aspect of the intrinsic noise. Our work emphasises a new approach to assessing sensory noise and provides the first complete description and evaluation of the relative impact of noise in phototransduction that does not rely on continuity, linearity or Gaussian approximations.

## Introduction

### The intrinsic-extrinsic relative noise problem in phototransduction

The phototransduction cascade consists of a series of chemical reactions which convert light inputs into usable current signals at the retina. As it serves as the front end to the visual system, it should be capable of extracting information from environmental inputs with high accuracy [1]. However, the cascade mechanism involves small numbers of molecules and therefore randomly executing reactions. Consequently, the electrical representation of each photon is non-identical and subject to inevitable variability. The signal is therefore degraded during photoconversion and the cascade said to be intrinsically noisy [2]. However, the cascade is not responsible for all the signal variability measurable at the retina. The light input itself introduces randomness through the variable timing of photons. Since the cascade has single photon sensitivity [3] this extrinsically introduced noise is also transferred across the cascade. This paper focuses on disentangling the relative contributions of these various noise sources.

The noise fidelity of phototransduction is critical. According to the data processing inequality [4], the information obtainable from the output of this process cannot be improved upon by later neuronal computations. As a result, whatever limits cascade performance may also delimit overall visual performance. An important and as yet unresolved question has been the determination of the relative influence of extrinsic photon noise as opposed to the intrinsic degradation caused by cascade reactions in representing the light stimuli. Since photon noise falls with intensity the relative noise quantification is necessarily a function of light intensity [5]. If in certain intensity regimes photon noise dominates, then the cascade can be considered sufficiently reliable for purpose at these luminance settings. If at other intensities the cascade noise is found limiting then one can query what characteristic of the phototransduction machinery is fundamentally stopping further intrinsic noise reduction.

This relative noise problem remains unsolved not only in invertebrate vision but also in vertebrate rods [6], and has parallels in other sensory systems like olfaction. While there are important structural and dynamical differences between invertebrate vision and rods (fundamentally different types of electrical signalling despite similar cascades [7]) and olfaction (photons are replaced by odour molecules [8]) they are all characterised by an overall motif known as G protein signalling. Hence, clarifying the invertebrate intrinsic-extrinsic noise relation can have useful and far reaching consequences for sensory analysis.

Previous researchers have focussed on quantifying noise limits by estimating the comparative contributions of photon and cascade noise on the electrical signal variance [9] or by applying linearised filter approximations to the cascade to calculate relevant noise spectra [10]. However, such approaches generally do not account for the point process or time ordered nature of this problem. These unmodelled problem dynamics may be especially important in phototransduction given that it is sensitive to individual photons and that its output is used in real time higher visual processing.

This paper will address these issues by applying and adapting Snyder filtering techniques [11] to data generated from the archetypal phototransduction system model of Drosophila melongaster. The Snyder filter is the point process analogue of the well known Kalman filter [12]. It provides a mathematically natural way of obtaining causal minimum mean squared error estimates of hidden Markov variables signalled via discrete observation events. Algorithms for extracting data from transduced photons will be developed and used in conjunction with Snyder based stimuli reconstruction techniques to show that photon noise dominates in low light while mean cascade delay limits system performance under bright conditions. This work will clarify and decompose the noise sources which dominate phototransduction while highlighting the importance of keeping the analysis discrete and causal.

### Discrete cascade dynamics define the intrinsic noise sources

The invertebrate phototransduction cascade consists of a series of stochastic molecular reactions. The Drosophila cascade is particularly important as an analysable and testable archetype, not only for invertebrate phototransduction, but also for more general G protein signalling motifs [13]. G proteins are important signal transducing proteins that are ubiquitous across many biological processes. It is therefore critical to understand the properties and performance of the Drosophila cascade. Such an understanding would not only provide general insight into major biological signalling strategies but could also contribute useful theory, applicable to artificial visual systems aiming at high performance with minimal processing.

Early vision in Drosophila involves light impinging on the rhabdomere of a photoreceptor, which is composed of about 30,000 microvilli [13]. Microvilli are essentially semi-autonomous processing units which absorb photons locally and produce quantum bumps (QBs) in response. The reaction set occurring in each microvilli involves photon absorption by Rhodopsin which leads to activation of the G protein via a guanosine diphosphate to guanosine triphosphate exchange. This results in activation of phospholipase C which liberates secondary messengers. There are only a few G protein and phospholipase C molecules per microvillus [14]. The secondary messengers activate the first light sensitive channel after a variable delay (15-100ms) [13]. This delay corresponds to the time required for the messengers to cooperatively overcome an effective channel activation threshold.

Activation of a single transient receptor potential (TRP) or TRP-like ion channel results in an influx of Ca^2+^ which rapidly activates the remaining channels in the microvilli (15-20 total) via a positive feedback mechanism. This generates a current which is quickly deactivated via a negative feedback Ca^2+^ based loop [13]. The effect of these regulated loops is the production of an essentially discrete, unitary photon signal or QB. A single QB codes for a single absorbed photon at a microvillus. The QBs generated by each stimulated microvillus sum linearly to produce the macroscopic photoreceptor response at the receptor cell membrane. As light intensity increases the QBs become faster and smaller (adaptation) which ensures that the cell dynamic range is properly utilised. Despite these changes it appears that photoreceptors are linear event counters even at daylight luminance values [14].

The entire cascade, from input photon to output QB is therefore described by transformations involving small numbers of discrete molecules or channels. This results in randomly timed reactions and probabilistic interactions that lead to process variability that is visible in the resulting QB signal [14]. The QB is a non-linear and stochastic electrical depolarisation that codes for an absorbed photon. Each QB has non-identical and randomly distributed latency, amplitude and duration. These stochastic QBs lead to variable representations of identical light stimuli which reduces the reliability of event representation. The variable latency between photon absorption and bump signalling is controlled by the time required for the second messenger to accumulate beyond a dynamic threshold [15]. The variable amplitude and width of the QB are determined by the sequential Ca^2+^ feedback loops. Evidence suggests that the QB latency is uncorrelated with the QB waveform and highly supports the conclusion that different and independent cascade reaction sets are responsible for these characteristics [16]. Consequently, one can think of QB shape distortion and latency as two independent and important manifestations of mechanistic cascade noise.

However, these sources of randomness, which are collectively termed cascade noise, are not the only forms of intrinsic noise [17]. Spontaneous (in the absence of light) activation of G proteins, Rhodopsin or single TRP channels can lead to spurious (false positive) QBs which are indistinguishable from photon stimulated QBs. While a possible issue at high temperature-low intensity settings, this dark noise source is negligible under the conditions investigated here and often the cascade suppresses these events via inbuilt molecular threshold signalling techniques [15]. Additionally a conceptual equivalent to false negative QBs also exists. When a light stimuli is presented to the cascade, microvilli respond independently to incident photons and produce respective QBs. Due to cascade stochasticity (random reaction timings), not every photon becomes a QB but instead there is a quantum capture efficiency, $\text{QE}\;=\frac{\text{no}.\;\text{effective}\;\text{photons}}{\text{no}.\;\text{incident}\;\text{photons}}$. Experiments, however, indicate that often QE is very high especially at low intensities where it can be close to 1 [18]. Hence the terms intrinsic and cascade noise will be used synonymously, and QE = 1 assumed. Supplements S3 Text, S4 and S5 Figs show that QEs of at least 0.66 have negligible noise impact.

Since there is a clear and intimate link between intrinsic noise and the cascade machinery, any noise analysis will require the incorporation of a physiological model that describes the full reaction set from Rhodopsin absorption to TRP channel opening. This work makes use of the Drosophila phototransduction model developed by Nikolic *et al* [19]. This model simulates all known reactions and mechanisms of fly phototransduction within a discrete, Poisson framework, uses known biochemical parameter estimates, and allows for multi-photon inputs. It was particularly chosen for this analysis since it provides a complete stochastic simulation of the cascade reactions, does not use common mass action (continuity) approximations and attempts to maintain a rich and unsimplified dynamical description of the process. Most importantly, it places emphasis on getting the noise distributions correct and in keeping with experimental data. Further, the model can be modified to account for other G protein based cascades, thus providing flexibility for future work [20]. Further information on the Nikolic model, including a mathematical description and a visualisation of the intrinsic noise component distributions, are provided in Supplements S4 Text, S7 and S8 Figs.

### Previous approaches neglect causality and system discreteness

This paper will investigate the relative noise source problem and quantify the main limitations on the reliability of early vision. Previous work on this problem has taken two main viewpoints. The first uses deviations from Poisson statistics as a measure of noise contributions. For a constant light intensity, photon absorption follows a Poisson distribution so that photon noise results in a photon catch variance to mean ratio of 1. If the measured ratio is above 1 then any excess variance must be due to intrinsic noise [9]. The results of this analysis suggest that at low intensities cascade noise contributed 50% and at high intensities 90% of all noise. This method provides a clear scheme for assessing relative noise sources that is easily interpretable. Unfortunately, it is not easily generalised to non-constant or pseudorandom light sources and depends on the intensity being low enough so that QBs are easily distinguishable (countable). Moreover, this scheme neglects causality which asserts that the phototransduced output at any time cannot depend on inputs after that time. Failing to factor in this information constraint can lead to spurious performance evaluations [21].

The second posits that the cascade aims to maximise (mutual) information transfer and calculates signal to noise ratios and channel capacities under a noisy, dynamically range-limited Gaussian channel assumption [22]. Relative noise sources are then described by how much they alter these ratios and are treated additively through their spectral densities [23]. This approach, by keeping analysis within the frequency-domain is analytically tractable and affords a convenient dissection of optimal sensory noise filtering. However, neither is the noise additive [24] nor neuronal dynamics Gaussian [25], making these methods at best approximate. Additionally, photons are point events and their resulting QB streams are countable sums of impulse responses. As a result the phototransduction system is necessarily discrete and the transfer of information from the environment to the retina is actually via a Poisson channel. This insight is a key motivator for the approach taken in this work. The stark coding differences between Poisson and Gaussian channels were noted by Ghanem *et al* in [26] and suggest that conducting the phototransduction analysis under a Gaussian (or continuity) assumption can be misleading. Additionally, these mutual information based studies neglect time order and use random variable based definitions of capacity [4]. In reality, the noisy phototransduction input is a stochastic process and the causal capacity should be expressed as in Lestas *et al* [21] instead. Consequently, these signal to noise ratio based schemes are probably only reliable at very high intensities where Poisson-Gaussian approximations are valid, and likely break down at lower luminance values, where the discrete nature of the system dominates.

In contrast to the above methods, this work directly and explicitly treats the time ordered, discrete and stochastic nature of the relative noise problem with point processes, integrate-fire algorithms and causal filters. No continuity or linearity approximations are made and a causal mean squared error distortion is used as the noise performance measure; in place of the usual signal to noise or variance to mean ratios. Getting the performance metric right is important, because application of different measures, even on the same data set, as noted in the work of Grewe *et al* [27], can lead to different and often misleading results. Since phototransduction should maximally preserve environmental information, using causal capacity based metrics would seem natural. However, embedding causality in this way is difficult. It is known from information theory that distortion functions (which directly calculate estimation error) provide a useful dual to capacity [4]. In this interpretation the concept of maximising information transfer is replaced with that of minimising distortion between a true and estimated stimuli. The distortion quantifies the difference between the true stimuli, denoted *x*(*t*) and some estimator of *x*(*t*). Note that *x*(*t*) is also referred to as the hidden state. Discreteness and causality are then directly accounted for by choosing an estimate that incorporates the naturally ordered and point-based structure that information takes when transmitted across a Poisson channel.

This work chooses the causal conditional mean as a suitable estimator. If the causal information available until time *t* are the observations $D_{0}^{t}$ then this estimator is $\hat{x}\left(t\right)=E[x\left(t\right)\;|\;D_{0}^{t}]$. This expectation integrates *x*(*t*) with respect to its conditional distribution given the causal data. The conditional mean estimator has a fundamental link to the ordered mutual information across a Poisson channel [28] and minimises a broad class of distortion functions. Among these is the function: $E\left[{\left(x\left(t\right)-\hat{x}\left(t\right)\right)}^{2}\right]$ (the expectation is now over state trajectories). This distortion is termed the minimum mean squared error (MMSE) and is chosen as the performance metric for this work. If some other estimator is used instead then this distortion is simply called the mean squared error (MSE). Any estimate which is a function of $D_{0}^{t}$ alone will incorporate causality and discreteness. However, it is only when it takes the form of $\hat{x}\left(t\right)$, the conditional mean, that it will minimise the MSE. Note that these square error distortions provide the additional advantage of not being sensitive to the removal of constant signals, which is a characteristic of the adaptation response in phototransduction [29]. Consequently the cleanest approach, involving the least approximations is to optimally and causally reconstruct the input intensity stimuli and directly calculate the MMSE. While mathematically more difficult than the Gaussian and variance approaches, comparing MMSEs between light intensity reconstructions subject to various noise sources is the most appropriate and quantifiable way of measuring relative noise contribution.

The importance of the approach presented here may extend beyond the phototransduction noise problem. Any real-time system which receives information sequentially and in packets is subject to the constraints of causality and discreteness. Developing and adapting techniques that can incorporate these often unmodelled dynamics can therefore have wide ranging importance. Accounting for these constraints has appeared as a concern, explicitly in molecular biochemistry [21] [30], and implicitly in general neuroscience [31]. In both cases information can be interpreted as being transferred over timing (Poisson) channels, with packets representing either molecular events or spikes and the real time estimation problem involving either molecular population inference or neuronal stimuli reconstruction. This work sits precisely within this framework.

Bobrowski *et al* [32] is the only other work (to the authors’ knowledge) to have (implicitly) dealt with causality and dicreteness. They showed that causal Snyder filtering could be used to obtain real time MMSE reconstructions of neuronal stimuli without the need for common approximations such as time discretisation. Using these techniques they estimated noisy, dynamic stimuli from discrete spiking streams. This paper extends their work by i) developing algorithms for the causal estimation of dynamic stimuli that are now no longer observable through the Poisson events they modulate, but must instead be inferred from noisy and potentially continuous waveforms based on those events, and ii) explicitly showing why factoring discreteness, non-linearity and causality is important for relative noise analysis. In this setting photons are interpreted as analogues to information bearing spikes and it is shown that is is possible to estimate complex light inputs from noisy outputs without the need for common linearity, continuity or Gaussian approximations.

### Markov-Poisson light models and physiological simulators are combined to solve a fundamental estimation problem

Since the relative noise problem quantifies the contributions of both extrinsic and intrinsic noise, it requires both an environmental light model and a phototransduction simulator. Visual neuroscience literature has often taken two approaches to light inputs (both in experiments and theoretical analyses) when investigating sensory performance [33]. The first is to repeatedly present simple inputs, which are easy to generate and control (for example short, constant intensity flashes), in the hope that they elicit simple responses that allow more transparent analysis of the sensory system. Averaging over the repetitions should then lead to meaningful results. The second involves using more complex, non-repeated stimuli that represent input forms that the sensory system is likely to encounter in normal operation. These naturalistic stimuli should reveal more intricate properties of the system as it is supposedly evolutionarily designed for such inputs. The downside of these is the loss of analytical tractability [33].

This works attempts to meld the advantages of both approaches by using simple but non-trivial, non-repeated light models that share some of the characteristics of naturalistic stimuli yet are still amenable to analysis. Specifically, a flickering, continuous time, discrete space Markov process description for environmental light intensity, λ(*t*) is used. This intensity modulates a Poisson process that produces a discrete photon stream, denoted as $P_{0}^{t}$ over the domain [0 *t*]. Time, *t*, is measured in ms with λ(*t*) having units of ms^−1^. These models are analytically treatable with Markov modulated Poisson theory. Naturalistic stimuli are known to have i) stochastic fluctuations which can be large, ii) relatively slow dynamics compared to the visual response time and iii) long correlation times [34]. The Markov modulated stimuli used here intrinsically possess these first two properties. The third property is approximated and simplified within the Markov assumption of the model.

There are two key differences, however, between the light models used here and the general naturalistic light stimuli often found in the literature. The first is that most stimuli are in terms of velocities and contrast and applied to motion detection (optic flow). The models in this paper are solely in terms of light intensity. Velocities and contrasts are not appropriate for this work as the paper treats light at a single photoreceptor so that no motion signal is present [35]. This property is therefore not relevant to this work. Secondly, naturalistic stimuli would likely encompass a broad range of intensity distributions depending on ambient conditions. As a result some of these would be dissimilar to those used here. However, given the rich range of intensity functions obtainable by appropriate choices of Markov parameters, this is not a true limitation. This work focuses on switching, bimodal stimuli. The fundamental formulation of this stimuli (called the interrupted model) is given in the models section of this paper. More complex and general bimodal light models are described in Supplement S2 Text.

This photon stream produced by the light model is the input to the phototransduction model. The Nikolic model described previously is used as the cascade simulator. It produces a QB output stream $Q_{0}^{t}$ which, at best, can only contain the information present in $P_{0}^{t}$ (by the data processing inequality [4]). By comparing estimates of the light intensity given $P_{0}^{t}$ or $Q_{0}^{t}$ one obtains an understanding of the noise deterioration at both the front and back end of the cascade respectively. To make the problem non-dimensional the normalised intensity, $x\left(t\right)=\frac{\lambda (t)}{\alpha }$, *α* > 0 ms^−1^ is used (it is explicitly described by the Markov states of the light model). The causal conditional mean estimates of *x*(*t*) given $P_{0}^{t}$, the raw photon stream, and $Q_{0}^{t}$, the quantum bump stream, are denoted ${\hat{x}}_{\text{ph}}\left(t\right)$ and ${\hat{x}}_{\text{qb}}\left(t\right)$ respectively. The comparison across the cascade is made with the MMSE indices: ${\text{mse}}_{\text{ph}}=E\left[{\left(x\left(t\right)-{\hat{x}}_{\text{ph}}\left(t\right)\right)}^{2}\right]$ which only measures photon noise (the noise floor) and ${\text{mse}}_{\text{qb}}=E\left[{\left(x\left(t\right)-{\hat{x}}_{\text{qb}}\left(t\right)\right)}^{2}\right]$ which characterises the combined impact of photon and cascade noise. These estimates of *x*(*t*) assume a spatial integration of the output from all stimulated photoreceptors. It is well know that QBs linearly sum over the low and medium intensity range up to hundreds of photons per second [36]. Consequently for the range of intensities investigated this assumption is valid.

Values of ${\hat{x}}_{\text{ph}}$ and mse_ph_ within this Poisson-Markov framework can be directly obtained from an optimal (MMSE) non-linear filter, known as the Snyder filter [11]. The Snyder filter is a Bayesian technique for inferring the posterior distribution of a hidden Markov state given modulated Poisson observations and a prior on the hidden states. Integrating this posterior gives the conditional mean estimator that is used in the MMSE distortion function. The Snyder filter is exact in that it makes no approximations or simplifications on either the state or observation dynamics. It solves a differential-difference equation on the state posterior leading to a deterministic continuous solution trajectory between observation (event) times with discontinuous updates at the observed random event points. The continuous component of the solution restarts after every event update and the overall posterior evolution falls within the framework of piecewise deterministic Markov processes. The equations behind the filter and their adaptation for this work are given in Supplement S1 Text. In general the Snyder filter does not require a Markov chain description of the state process. The inference procedure developed here may therefore be a useful blueprint for analyses involving other types of light models. For details of its general formulation and its application to other types of state estimation problems in biology see [11] and [32] [37] respectively.

In contrast, there is no known method for calculating ${\hat{x}}_{\text{qb}}$ and mse_qb_ since it is difficult to describe the cascade noise within a tractable analytical framework. This paper develops algorithms for estimating and bounding ${\hat{x}}_{\text{qb}}$ and mse_qb_. By comparing these estimates to ${\hat{x}}_{\text{ph}}$ and mse_ph_ meaningful conclusions will be drawn about the relative impact of intrinsic and extrinsic noise on the normalised intensity input.

## Models

### Fundamental Markov on-off light model

**Fig 1:**
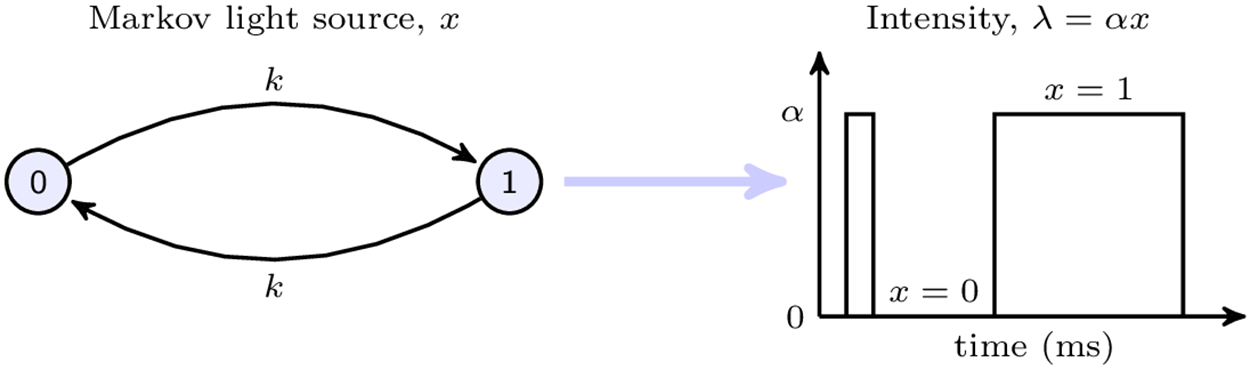
Markov on-off interrupted light model and intensity function. State 1 indicates the light is on and switching occurs with mean exponential waiting time of $\frac{1}{k}$. Photons are emitted with mean rate *αx*(*t*) with the model state *x*(*t*) ∈ {0, 1} and un-normalised intensity λ(*t*) ∈ {0, *α*}.

Two main Markov light models are used in this work. The first is a fundamental, symmetric, stochastic on-off light switch which emits photons according to an interrupted Poisson process. It is also known as the random telegraph signal. The second is a multi-state Markov model which has a bimodal Gaussian type state distribution and represents a complex light source that flickers between two extreme intensities while also possessing small light changes about the extreme modes. The bimodal model can be thought of as a generalisation of the interrupted model to higher state spaces with additional switch frequencies. The mathematical details of the former follow. The bimodal model and its qualitative similarities and differences to the interrupted source are described in Supplements S2 Text, S1, S2 and S3 Figs. The interrupted light source with state transition rates set at *k* > 0 ms^−1^ is shown in Fig 1. This model has the maximum entropy rate of any 2 state stationary Markov source and is therefore the most complex model (from an information theory perspective) for its state size [4].

To better compare and quantify the behaviour of light models, two dimensionless parameters are introduced. The first, $\beta =\frac{\alpha }{k}$, characterises the number of photons produced on average per on state and can be understood as a relative (normalised) light intensity descriptor. Higher *β* indicates more photons per state and hence more usable information. The second, $\gamma =\frac{k^{-1}}{100}$, measures the number of QBs (with a reference maximum width of 100ms) that can fit into the on time of the underlying Markov chain. This parameter basically describes the relative flicker of the light stimuli. Higher *γ* implies a relatively slower flickering light model, which should be easier to estimate. Thus, all light models will be compared in terms of their (*β*, *γ*) setting with increases in either likely to lead to better MSE. For more complex stimuli, such as the bimodal model, *k* is defined as the smallest death rate on the chain.

Given the stream of photons produced by the interrupted model, an appropriate Snyder filter can be formulated to obtain the posterior state distribution and then the conditional mean estimator. The Snyder filter description usually involves a coupled differential-difference equation for each Markov state posterior component. These dynamical equations are given in Supplement S1 Text. Since the interrupted model has only 2 states and probabilities must sum to 1, the complete Snyder equation set can be reduced to a single expression in terms of the conditional mean estimate ${\hat{x}}_{\text{ph}}$. When solved with the dummy variables *A* = *β*^−1^ + 2^−1^, $B=\sqrt{\beta^{-2}+2^{-2}}$ and $C=\tanh^{-1}(\frac{A-1}{B})$ and initial condition ${\hat{x}}_{\text{ph}}\left(0\right)=1$ (assumes a photon at *t* = 0) the following conditional state estimator results:

$$\begin{array}{cc} & {\hat{x}}_{\text{ph}}\left(t\right)=A-B\tanh\left(B\alpha t+C\right)\;\text{for}\;0\leq t\leq s^{-} \end{array}$$ (1)

$$\begin{array}{cc} & {\hat{x}}_{\text{ph}}\left(s^{+}\right)=1 \end{array}$$ (2)

The equations above are given until some time *s*, when a new photon arrives, with *s*^−^ and *s*^+^ indicating time infinitesimally before and after the photon. This hybrid solution provides useful insight into the behaviour of the optimal MMSE estimator. On every photon ${\hat{x}}_{\text{ph}}$ discontinuously jumps to a value of 1 (eq 2) and then continuously decays until the next photon arrives (eq 1) with a minimum possible value of $\lim_{t\to \infty }{\hat{x}}_{\text{ph}}\left(t\right)=A-B$ where 0 ≤ *A* − *B* ≤ 0.5. The estimator trajectory is reset on every event (local time becomes 0 again).

**Fig 2:**
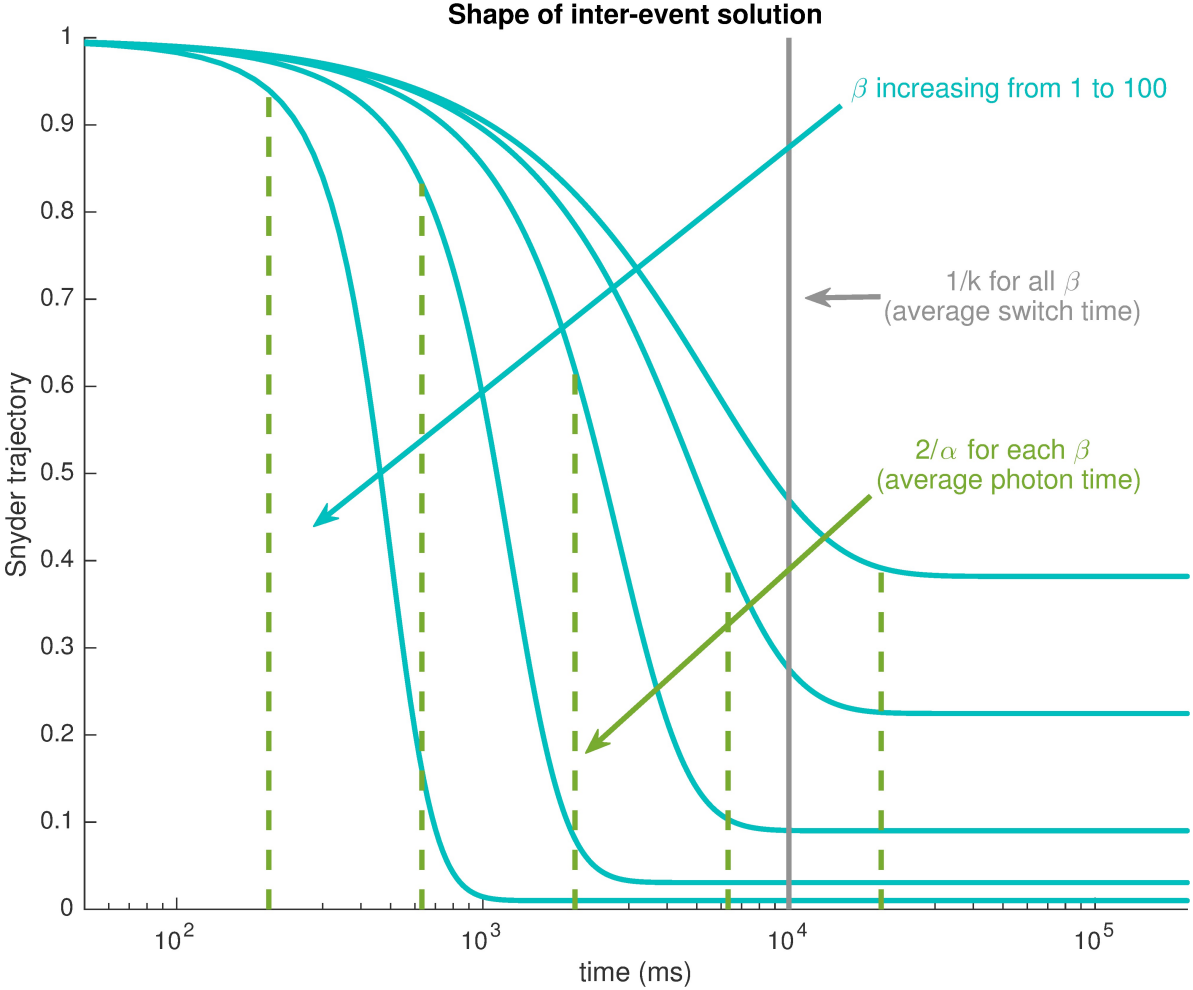
Snyder inter-event intensity estimate for the interrupted model when all photon times are known. The solution ${\hat{x}}_{\text{ph}}$ decays more rapidly relative to the switch time $\frac{1}{k}$ as the mean number of photons per on state of the light model, *β* increases. The solution stays close to state 1 with increasing *β*, relative to the time to the next photon $\frac{2}{\alpha }$. This indicates increased certainty as there is more information available.

The inter-event solution at different *β* is given in Fig 2. For the interrupted model, the mean time between photons is $\frac{2}{\alpha }$ ms [38], while the mean time between on-off switches is $\frac{1}{k}$ ms. Higher *β* implies more available information and lower mse_ph_ values. This effect is observable in the decay curves of ${\hat{x}}_{\text{ph}}\left(t\right)$. At low *β*, due to less certainty about the state, the scheme relatively quickly decays to a steady value approaching 0.5. As *β* → 0, ${\hat{x}}_{\text{ph}}\left(t\right)\to A-B=0.5$, and mse_ph_ → 0.25. This is no better than simply choosing the best constant estimate of ${\hat{x}}_{\text{ph}}\left(t\right)=E\left[x\left(t\right)\right]$. At high *β* the decay is slower (relative to the normalisation time $\frac{2}{\alpha }$) and the estimate stays close to 1 sufficiently long such that if another photon would occur their would be little decay between them. However, the decay is sufficiently fast relative to the switching time so that if no photons occur for an adequate amount of time then the quick fall to *A* − *B* ≈ 0 accurately reflects the state switch. This is especially true for very high *β* where photons essentially delineate the boundaries of the of the *x*(*t*) trajectory such that staying close to 1 between photons and decaying to 0 in the absence of photons will, as *β* → ∞, result in mse_ph_ = 0. The more generalised filter solution for any Markov model and an illustration of the resulting trajectory for the bimodal model can be found in Supplements S2 Text and S2 Fig respectively.

## Results

### The integrate-fire-Snyder algorithm converts bumps into estimated photon times for Snyder filtering

While the Snyder filter computes the optimal estimate ${\hat{x}}_{\text{ph}}$ exactly, there is no known equivalent method for obtaining ${\hat{x}}_{\text{qb}}$, the MMSE estimator given the bumps. One contribution of this paper is an algorithm that allows calculation of a good substitute for ${\hat{x}}_{\text{qb}}$, denoted ${\hat{x}}_{\text{qb}}^{*}$, using the QB data stream. This non-MMSE estimator would then provide a useful upper bound on the combined distortion resulting from both intrinsic and extrinsic noise. The known relationship of the estimators can then be summarised as ${\text{mse}}_{\text{ph}}\leq {\text{mse}}_{\text{qb}}\leq {\text{mse}}_{\text{qb}}^{*}$. The quantity ${\text{mse}}_{\text{qb}}^{*}-{\text{mse}}_{\text{ph}}$ upper bounds the noise introduced by the cascade since mse_ph_ is precisely the distortion due only to the photon input variability.

The scheme developed here for deriving ${\hat{x}}_{\text{qb}}^{*}\left(t\right)$ is called the integrate-fire-Snyder algorithm. The integrate-fire part of the name reflects its similarity to a standard neuronal model [39]. Consider a summed QB stream $Q_{0}^{T}$ for some maximum observation time, *T*, obtained by applying the photon stream $P_{0}^{T}$ to the Nikolic phototransduction simulator [19]. The algorithm uses $Q_{0}^{T}$ to first derive an estimated photon stream ${\hat{P}}_{0}^{T}$. This is then applied to the Snyder filter to obtain ${\hat{x}}_{\text{qb}}^{*}\left(t\right)$. While the standard Snyder filter cannot achieve MMSE estimates given a distorted photon stream, as long as ${\hat{P}}_{0}^{T}$ is sufficiently close to $P_{0}^{T}$ in some sense then ${\text{mse}}_{\text{qb}}^{*}$ should not be too much larger than mse_qb_. The integrate-fire part of the algorithm generates a good ${\hat{P}}_{0}^{T}$ from the QB signal by minimising the dissimilarity between the real and estimated photon streams. This is done by treating photons as spikes and optimising a spike distance metric across a training data set. The Victor-Purpura D^spike^ [40] measure, which calculates the optimal transformation from one train to the other via shifts, deletions or insertions of spikes (all of which have an associated transformation cost), was used. D^spike^ performed well and possessed clear minima. In contrast, optimising directly using the calculated ${\text{mse}}_{\text{qb}}^{*}$ was found to be computationally problematic.

Let $P_{0}^{T}$ and $Q_{0}^{T}$ be partitioned into training sets *P*_*a*_ and *Q*_*a*_ and test sets *P*_*b*_ and *Q*_*b*_. The training set is used to optimise the mapping from the real photon stream to the estimated one. The test set ensures the designed mapping properly captures the overall relationship and is neither over-trained nor under-trained. The training stage optimises a constant $\zeta =m\frac{\int Q_{a}dt}{\int P_{a}\;dt}$ where the integral is over the time frame of the training set and *m* is the varying parameter of interest. Here *ζ* is interpreted as a multiple of the average charge integral of a single photon QB response. The estimated time of the *n*^th^ photon in the testing stage occurs at the first time *t*: ∫*Q*_*b*_ *dt* > *nζ*, with the integral over the test data set. The method fires photons every time the integrated QB signal crosses an optimised threshold, *ζ*.

**Fig 3:**
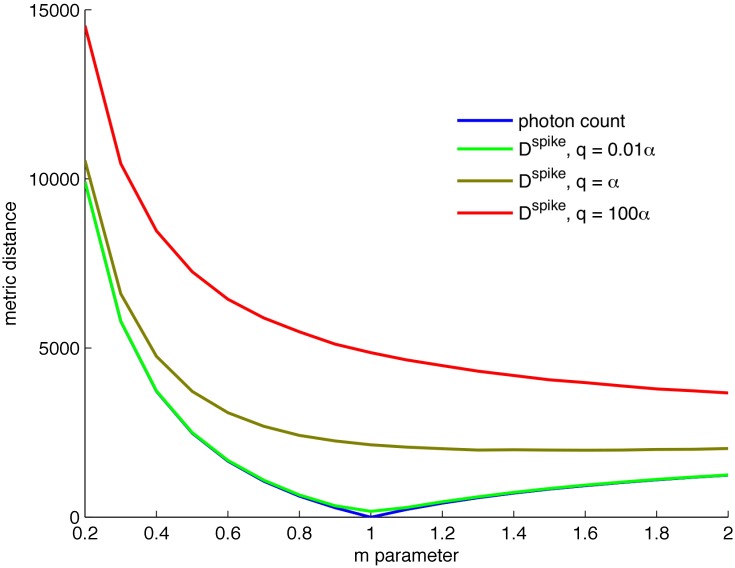
Spike metric optimisation shows a clear minimum when only photon counts are used. Parameter *q* indicates the relative cost of shifting versus inserting/deleting photons. For small values of *q* (shifts are unimportant) a clear minimum appears at *m* = 1 indicating integrate-fire works best with a threshold set at the mean bump area.

Fig 3 shows the spike metric optimisation curves, which have a clear minimum at *m* ≈ 1. The best ${\text{mse}}_{\text{qb}}^{*}$ values were obtained when the shift cost of D^spike^ goes to 0. At this setting the metric gives the difference in the photon count between the real and estimated stream. This can be confirmed by defining a photon count cost on the training set as $\mu =|\int P_{a}\;dt-\int {\hat{P}}_{a}\;dt|$. Here ∫*P*_*a*_ *dt* = 〈*Q*_*a*_〉^−1^∫*Q*_*a*_ *dt* is the integral of the true photon stream from the training set and $\int {\hat{P}}_{a}dt=m{\langle Q_{a}\rangle }^{-1}\int Q_{a}dt$ is the integral of the estimated photon stream using the integrate-fire method with parameter *m*. The quantity 〈*Q*_*a*_〉 = *ζm*^−1^ is the average QB charge per photon, from the training set. Substituting these expressions: *μ* = (1 − *m*)*mζ*^−1^∫*Q*_*a*_ *dt*. This is minimised when *m* = 1 for any QB set. This optimisation will therefore produce good results as long as the QB charge per photon is similar in the training and test sets. This condition is guaranteed for sufficiently large test and training sets. Thus, using the optimal *m* = 1, the integrate-fire converts some input photon stream into an estimated one every time ∫*Q*_*b*_ *dt* crosses an integer multiple of 〈*Q*_*a*_〉. The resulting stream is then fed into the Snyder filter to produce ${\hat{x}}_{\text{qb}}^{*}\left(t\right)$.

The use of a perfect integrator (with *m* = 1) may suggest that errors from previously processed QBs (due to their area deviating from average) would persist with observation time and influence the signalling of upcoming QBs. Often in neuronal modelling this error memory problem is solved by adding a term that continuously drains some of the accumulated charge. This makes the integrator ‘leaky’ [39]. However, given the variation of QB area around its mean size it appeared that these memory errors negated each other and lead to the inference of the correct number of photons. Since training with D^spike^ indicated that it was more important to get the number than the position of the inferred photons correct; perfect integration worked for this problem. It is not clear that making the scheme leaky would improve performance. If used naively, for example, then losing charge would lead to less effective photons being signalled (false negatives) which could deteriorate performance. However, it could improve performance if *m* and the leaky time constant, *ϵ*, are jointly optimised during training so that the number of photons signalled remains the same as in the perfect integrator case. The value of *ϵ* might then help get the positions of estimated photons closer to those of the real stream. This could only strengthen the following results by tightening the MSE upper bound on the true mse_qb_.

**Fig 4:**
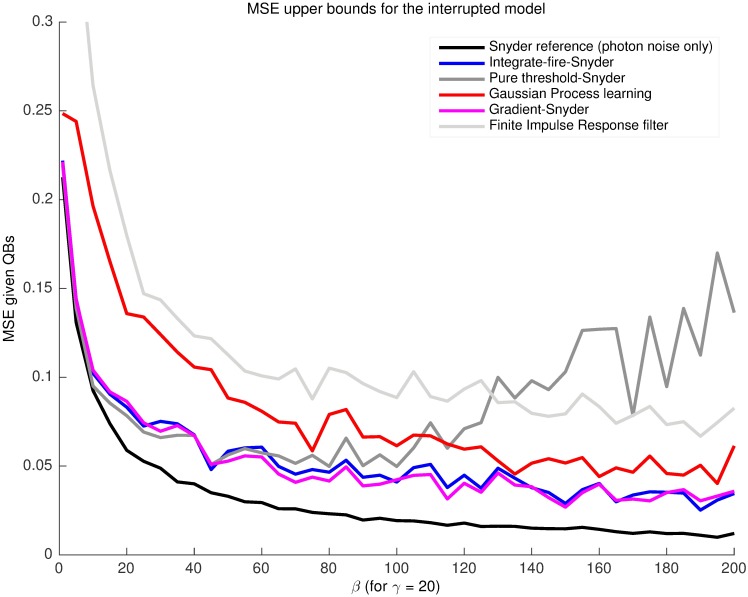
The integrate-fire-Snyder outperforms other machine learning and photon estimating schemes across intensity. The integrate-fire was tested against Gaussian processes and optimised finite impulse response filters which used the bump data directly. It was also compared to other schemes that also estimated photons for processing with Snyder filters. These fired photons based on pure current thresholds or bump gradients. Data is shown for the interrupted model at *γ* = 20 for a given representative light model trajectory of 7000-8000 photons. The integrate-fire showed superior overall performance. Consistent results have also been obtained for *γ* = 5. This motivated the use of the integrate-fire-Snyder as a cascade estimator.

**Fig 5:**
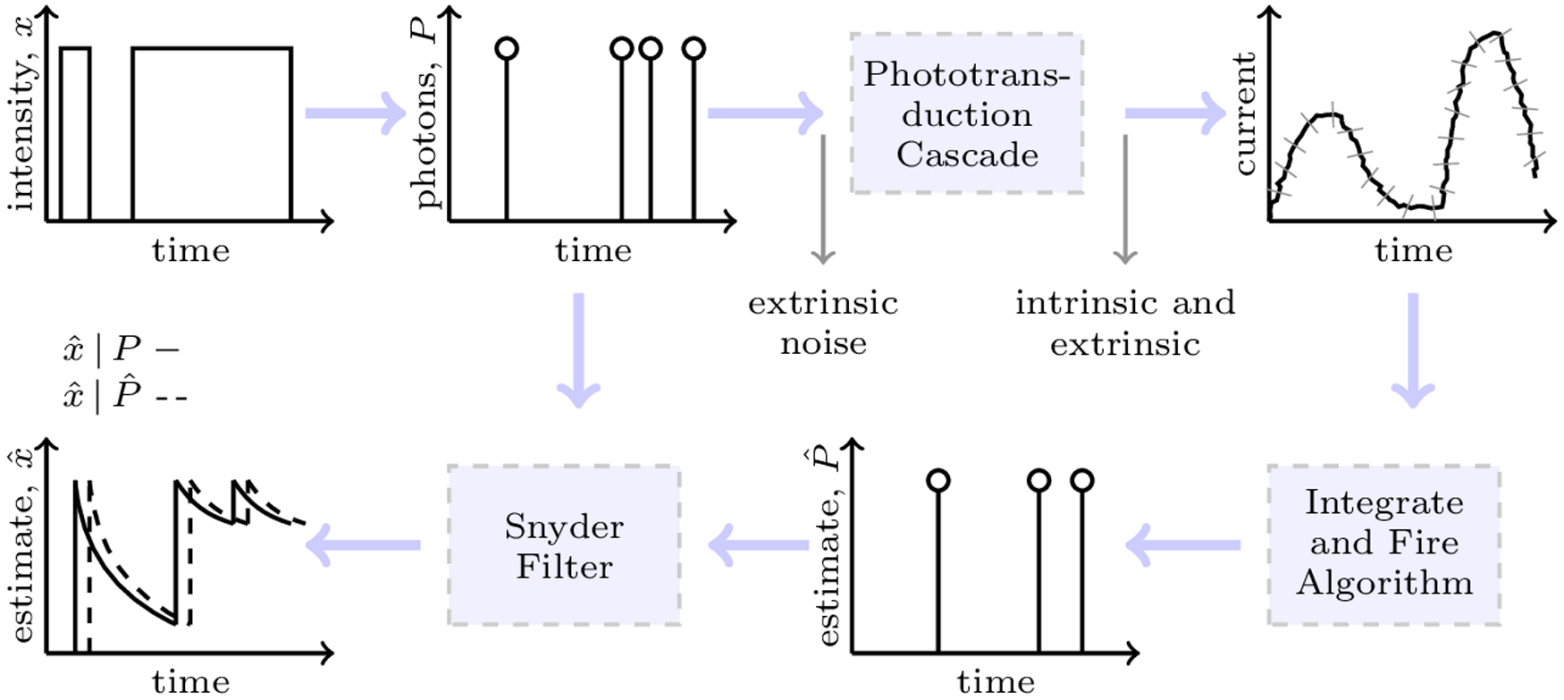
General integrate-fire-Snyder scheme. The standard Snyder filter gives the optimal (MMSE) intensity estimate given known photons. The random square wave intensity producing these photons delineates the on-off times in the interrupted light model. The MMSE estimate exactly characterises achievable performance at the front end of the cascade. The photons are converted into bumps by the cascade. The integrate-fire uses an optimised threshold to convert these bumps into estimated photons. These estimated photons are then Snyder filtered to obtain a non-MMSE estimator that upper bounds the deterioration introduced by the cascade.

The integrate-fire-Snyder scheme was compared to standard supervised machine learning techniques (Gaussian Processes [41]) and optimal linear filtering methods (least squares Finite impulse response [42]) which directly estimated a ${\hat{x}}_{\text{qb}}^{*}$ from the same $Q_{0}^{T}$. The former attempted to fit a covariance function while the latter minimised the square error on a series of filter coefficients. Both methods used variable window sizes (also known as fading memory, this describes how much of the input history affects the output) to get the best performance. These methods worked directly on the QB data without estimating photons. The optimal window size for both schemes was found to be about $5(\frac{2}{\alpha })$. A representative simulation of the resulting MSE upper bounds from these techniques is given in Fig 4. The integrate-fire achieved a lower ${\text{mse}}_{\text{qb}}^{*}$ than all of these machine learning schemes. Thus, it appears that estimating a photon stream and applying the Snyder filter leads to appreciably better results than methods which directly estimate *x*(*t*) from the QB waveform. This figure also shows the relationship between the integrate-fire estimator and other techniques which also estimated photons for use with a Snyder filter. The ‘Pure threshold’ scheme produces estimated photons whenever a simple electrical signal threshold is exceeded and the ‘Gradient’ one does so when a forward and backward gradient shift is observed on the QB waveform. The integrate-fire outperformed the threshold scheme but had comparable MSE to the gradient one. However, the gradient scheme is not a robust technique, making integrate-fire the best overall choice. An overview of the general integrate-fire-Snyder estimation process is given in Fig 5.

### Optimal linear and optimal Snyder filtering on delayed photons and their performance relative to integrate-fire-Snyder filtering

To illustrate the importance of using non-linear filtering techniques for this analysis, consider the optimal (MMSE) linear estimate given $P_{0}^{T}$, denoted ${\hat{x}}_{\text{ph}}^{l}$, with distortion ${\text{mse}}_{\text{ph}}^{l}$. For the interrupted model, this linear MMSE is obtained by applying a continuity approximation to the system state dynamics [43] [44]. This leads to a differential equation with steady state solution (see Supplement S1 Text):

$$\begin{array}{cc} & {\text{mse}}_{\text{ph}}^{l}=\frac{1}{\beta }(\sqrt{1+\frac{\beta }{2}}-1) \end{array}$$ (3)

**Fig 6:**
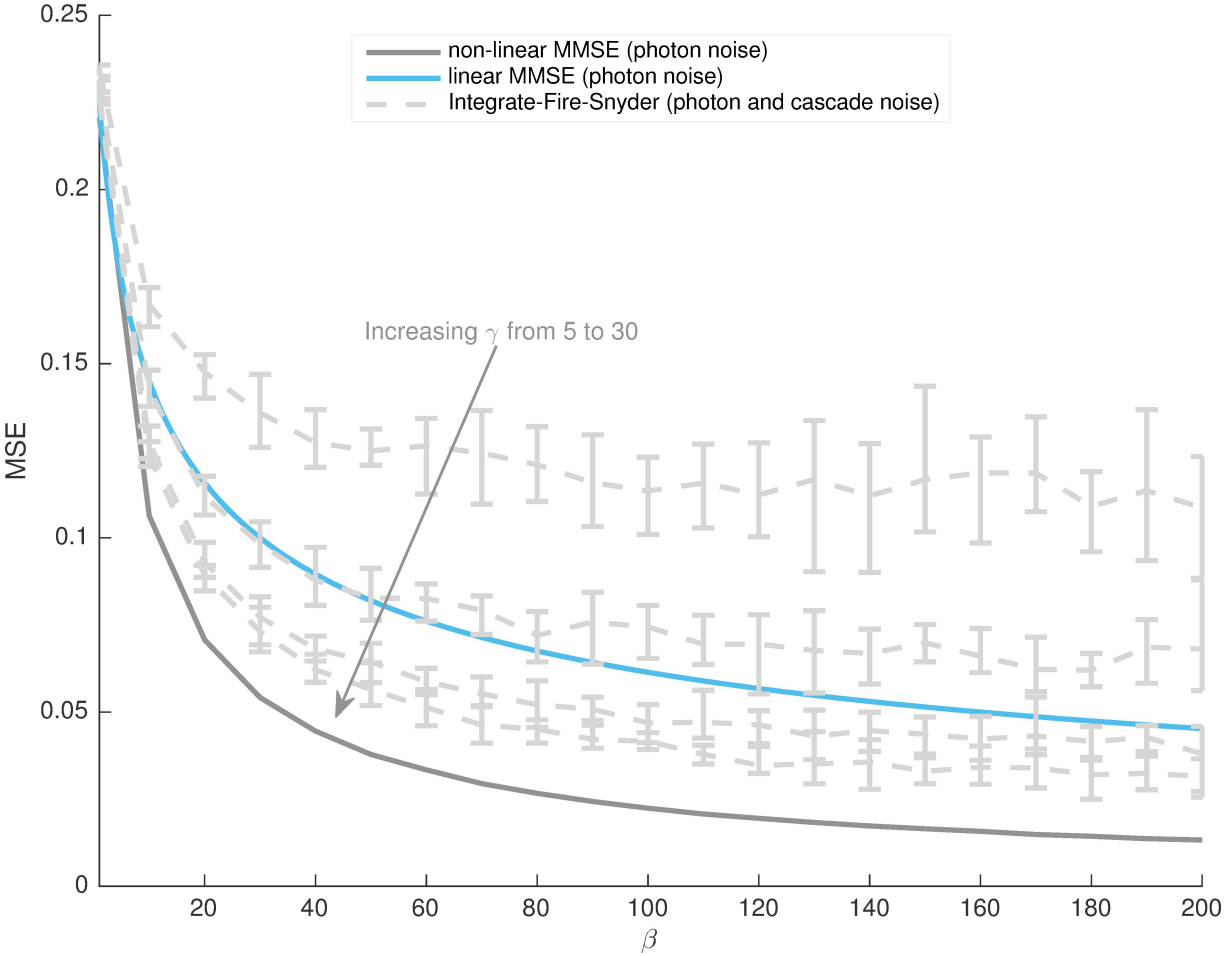
Suboptimal integrate-fire-Snyder on bumps outperforms MMSE linear filtering on known photons. The integrate-fire-Snyder was run for *γ* = [5, 10, 20, 30] and results across 10 independent 8000 photon long light model trajectories given. The error-bars delineate the minimum to maximum of the MSE curves for each *γ*. Clearly there exist several *γ* over which the integrate-fire on all cascade noise does better than the linear MMSE on just photon noise, across a wide *β* range. Given the disparity, applying linear or continuity approximations can be a strongly misleading measure of the noise performance of discrete stochastic systems.

The central importance of the non-dimensional parameter *β* is now obvious. Comparison of the optimal linear estimate given true photons, ${\text{mse}}_{\text{ph}}^{l}$, the optimal non-linear estimate given the true photons, mse_ph_, and the integrate-fire-Snyder estimate (at several *γ*) from the resulting bumps, ${\text{mse}}_{\text{qb}}^{*}$, are given in Fig 6. The significant difference between ${\text{mse}}_{\text{ph}}^{l}$ and mse_ph_ indicates that some of the MSE is being contributed by the inefficiency of the linear filter as an estimator (since it does not fully model the system dynamics). This additional error can likely cloud or misrepresent the true relative impact of different noise sources. The fact that ${\text{mse}}_{\text{ph}}^{l}$ is above ${\text{mse}}_{\text{qb}}^{*}$ at several *γ* suggests that these approximations reduce the ability to resolve the more subtle contributions of cascade variability. Consequently, linearity or continuity approximations can seriously distort one’s view of phototransduction noise performance.

As concluded above, keeping analysis exact and non-linear is necessary to properly resolve the contributions of various cascade noise sources. Comparing the integrate-fire-Snyder results with filtering solutions that only feature specific components of the cascade noise should achieve this resolution. Such analysis allows one to identify the major (independent) intrinsic noise sources clearly. These mainly include QB shape (width and height) variation and latency noise (see Introduction). The impact of shape noise was assessed by elimination and the application of the integrate-fire-Snyder to a deterministic version of the Nikolic model that featured QBs of fixed size and shape. More detail on the differences between stochastic and deterministic Nikolic models is given in Supplement S4 Text.

QB response latency can be decomposed into a (deterministic) mean delay and a jitter term describing variation around the mean. The effect of the total QB latency was isolated by applying the Snyder filter to photons that have been empirically delayed according to the physiological latency distribution embedded within the Nikolic model. The empirically delayed MSE upper bounds are given in Supplements S4 and S5 Figs. To quantify the relative impact of jitter and mean delay a Snyder filter that optimally reconstructs light intensity given a deterministically delayed photon stream was developed.

If the original photon stream up to time *t* is $P_{0}^{t}$, then denote the delayed stream $P_{0}^{t-\tau }$ as $M_{0}^{t}$ with delay *τ* ms. The optimal filtering problem focuses on finding the posterior probability vector $\text{P}(x\left(t\right)|M_{0}^{t})$. Let the conditional estimate ${\hat{x}}_{d}\left(t\right)=E[x\left(t\right)|M_{0}^{t}]$. Since introduction of the delay does not alter the inter-event spacing of the observed events then the usual Snyder filter can be applied to obtain the estimate $\hat{x}\left(t-\tau \right)$ using $M_{0}^{t}$ as the observed process. Transforming $\hat{x}\left(t-\tau \right)$ into the desired optimal estimate ${\hat{x}}_{d}\left(t\right)$ is possible using $\text{P}(x\left(t-\tau \right)|M_{0}^{t})$, which is produced by the original Snyder filter. Since there is no data (no observed events) the evolution from the delayed to the desired posterior is achieved using the Chapman-Kolmogorov equations, with initial condition *t* − *τ*. *R* describes the transition rates of the *x*(*t*) Markov chain (see Supplement S1 Text for more details). This gives $\text{P}\left(x\left(t\right)|M_{0}^{t}\right)=\text{P}\left(x\left(t-\tau \right)|M_{0}^{t}\right)e^{\tau R}$ which is used to calculate the expectation ${\hat{x}}_{d}\left(t\right)$. The solution for the interrupted model between and at event times is provided in eqs 4 and 5 respectively.

$$\begin{array}{cc} & {\hat{x}}_{d}\left(t\right)=\frac{1}{2}(1-e^{-2\tau k})+\hat{x}\left(t-\tau \right)e^{-2\tau k}\;\text{for}\;0\leq t\leq s^{+} \end{array}$$ (4)

$$\begin{array}{cc} & {\hat{x}}_{d}\left(s^{+}\right)=\frac{1}{2}\left(1+e^{-2\tau k}\right)<1\;\text{if}\;\tau >0 \end{array}$$ (5)

This solution is optimal and not an upper bound like the integrate-fire-Snyder ${\text{mse}}_{\text{qb}}^{*}$ curves. When compared to the standard Snyder solution of eqs (1) and (2), the delayed solution features a higher minima and a lower maxima. This shows the increased uncertainty in this problem as the solution lies closer to the uninformed estimator E[x(t)]=0.5. Observe that $\lim_{\beta \to 0}\;{\hat{x}}_{d}\left(t\right)=\lim_{\beta \to 0}\;{\hat{x}}_{\text{ph}}\left(t\right)=0.5$.

### Comparison of various reconstruction estimates

**Fig 7:**
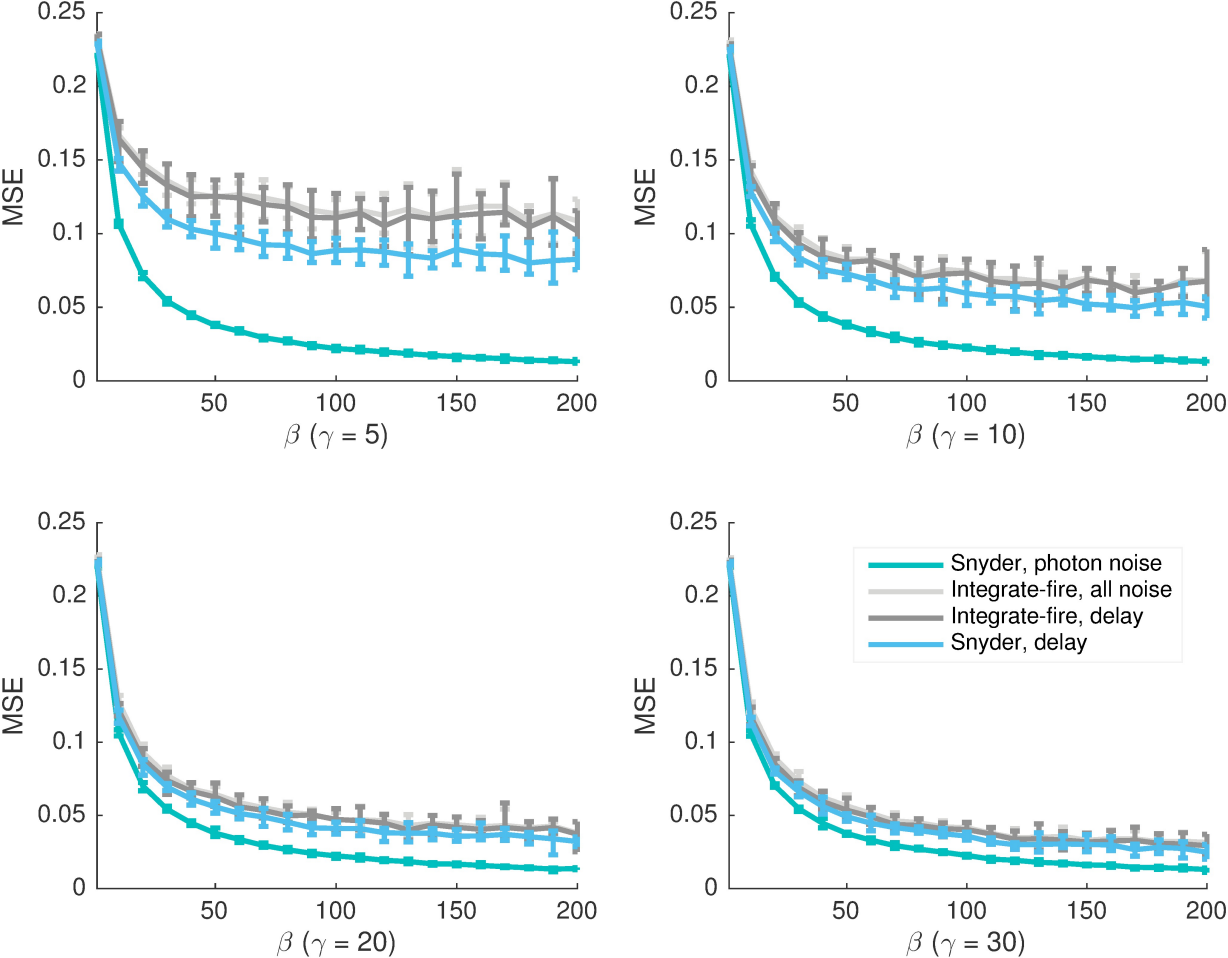
The dominant noise transition from extrinsic to mean delay, with intensity *β*, is consistent for light models with differing relative flicker speed, *γ*. Parameter *γ* indicates the number of bumps which can be received per Markov switch time of the light model. Higher *γ* therefore means a relatively slower flicker. At lower *γ* less information is available and hence the MSE higher. The ‘Snyder, delay’ curves are MMSE values obtained with the Snyder filter optimised for a deterministic delay set to the mean QB latency. These lower bound the noise introduced by the cascade. The ‘Integrate-fire, all noise’ ones are upper bounds as they involve processing the QBs from the fully stochastic Nikolic model. The closeness of both of these curve sets reflects the dominance of mean delay among cascade noise components. This hypothesis is further confirmed by the closeness of ‘Integrate-fire, all noise’ with the MSE generated by applying the integrate-fire-Snyder on the deterministic Nikolic model, which only featured the mean delay (‘Integrate-fire, delay’) and fixed QB shapes. The ‘Snyder, photon noise’ gives the noise floor since it is the MMSE achievable at the front of the cascade. Convergence with this at low *β* shows cascade noise is unimportant in that regime. All curves are averages over 10 independent 8000 photon-QB streams with error bars indicating the maximum and minimum MSE swings around this value.

The main results from preceding sections are combined into Fig 7. Results are averaged over 10 independent photon-QB streams of 8000 photons long with error bars showing the maximum and minimum MSE across the runs. Flickering models at *γ* = [5, 10, 20, 30] are investigated. At *γ* > 30 the relative curve behaviour is unchanged and at *γ* < 5 the model switches too fast for sensible inference (it is beyond the bandwidth of the phototransduction process). The *β* range (1-200) was chosen similarly. Lower *β* means photons are produced too slowly relative to light switches making inference pointless (hence why MSE settles near 0.25) while higher *β* is not any further informative.

The ‘Snyder, photon noise’ curves give mse_ph_, or the noise floor of the model while ‘Snyder, delay’ refers to mse_d_ (photon noise and a deterministic delay). The remaining curves are integrate-fire-Snyder upper bounds. The ‘all noise’ ones correspond to ${\text{mse}}_{\text{qb}}^{*}$ (fully stochastic Nikolic model) while the ‘delay’ ones refer to the integrate-fire-Snyder applied to QBs from the deterministic Nikolic model. This last curve set is used to confirm the subsequent conclusions and involves applying the integrate-fire-Snyder algorithm to the Nikolic model with all its variability switched off. Consequently, the QB stream has deterministic bump shapes and sizes and there is no latency jitter. As a result, only the mean of the stochastic delay acts in this model. These 4 sets of curves provide an overall perspective of noise performance with relative light intensity and illustrate the main conclusions that will follow.

At low *β* all curves converge, while at higher *β* the cascade noise separates the ‘Snyder, photon noise’ ones from the rest. The ‘Snyder, photon noise’ curves are exactly the MMSEs achievable at the front of the cascade, and cannot be bettered. The ‘Snyder, delay’ curves are hard lower bounds, in the sense that they provide a lower limit on the remaining error when all sources of cascade variability are removed. Since this setting also applies to the deterministic Nikolic model then the ‘Integrate-fire, delay’ is an upper bound on the impact of the mean QB delay. The precise MMSE curve under this setting would lie between these bounds. The ‘Integrate-fire, all noise’ curves are an upper bound on all photon and cascade based distortion. All 3 of these sets of bounds remain close at higher *β* suggesting mean delay is the key intrinsic noise component. The relative behaviour of the MSE curves with *β*, remains unchanged as *γ* changes. Higher MSE values are observed at lower *γ* as a relatively faster flickering model (less QBs are available per state) is more difficult to estimate. In all simulations *τ* = 43.3 ms is used to match the mean delay from the Nikolic QB latency distribution.

This analysis was also repeated using more complex and realistic 16 state bimodal light models. Similar results were obtained and the relative behaviour of the MSE curves found to be consistent with the above descriptions (see Supplements S2 and S3 Text, S4 and S5 Figs). In the bimodal model higher frequency small amplitude fluctuations are superimposed on a lower frequency flickering (modal switching) between high and low light levels (the modes). This gives a characteristic $\frac{1}{f}$ like power spectrum behaviour over a frequency, *f*, range of almost two decades. The precise exponent was *f*^−1.23^.

### The integrate-fire-Snyder algorithm uses simple computations that are potentially biologically realisable

The integrate-fire-Snyder algorithm is a surprisingly simple yet effective way of reconstructing light intensity from the bumps. Given these attributes, one wonders if it could potentially be implemented within a biologically sensible neuronal network framework. The algorithm is composed of two main parts; i) an integrate-fire estimator that gives ${\hat{P}}_{0}^{t}$ and ii) the Snyder filter that leads to the state estimate ${\hat{x}}_{\text{qb}}^{*}\left(t\right)$. The integrate-fire part involves the accumulation of QB charge until some spike optimised threshold, *ζ* is crossed. Once *ζ* is exceeded a point event is emitted to indicate a suspected photon. This description is standard in neuronal modelling with the build up of charge usually leading to the release of an action potential or spike [39].

While the integrate-fire mechanism is biologically implementable, the optimisation of its threshold (which depends on the parameter *m*) requires a supervisory learning technique that uses true photon times. In reality, the invertebrate visual system does not have access to this data. Unsupervised learning procedures may therefore be necessary. If light intensity is low, unsupervised integrate-fire training should be easily realisable. In this regime QBs are distinct and far apart so the integration threshold is obvious. In the high intensity regime where QBs merge together, a more sophisticated way of reducing the data into effective photons is needed. This would likely involve finding clusters (which code for sets of QBs) within the data. Standard algorithms exist for extracting clusters and structure from input data. These can be implemented in modular, Hebbian learning networks [45] which are meant to resemble actual spiking neuronal computational behaviours. Hence, it should be possible to train the integrate-fire scheme in a biologically sensible manner, especially as only a single parameter must be learnt. However, given that threshold optimisation found that the integrate-fire should only signal photons every time the average bump charge is crossed, training may not be necessary. The average QB area could likely be a parameter that is naturally encoded in the cascade biology.

The second part of the algorithm uses standard Snyder filtering. The filter solution involves an instantaneous update at the event time which serves as the initial condition for a decaying inter-event trajectory. For all Markov chain state models it can be shown that the inter-event Snyder solution can be expressed as a linear equation set in un-normalised probabilities that must then be normalised [46] (see Supplement S2 Text). Using this formulation the inter-event trajectory can be seen as only involving weighted combinations of exponentially decaying functions. Bobrowski *et al* [32] provided strong evidence that these linear Snyder computations can be achieved with a recurrent neural network that is physiologically realisable and which uses simple operations such as weighted sums. This involves a graphical structure that treats incoming spikes as inputs from a sensory layer and encodes the causal posterior components, with another layer that has weights derived from the Markov state transition rates. Having a neuronal population code for posterior probabilities has already been shown to be biologically plausible [47]. Further, it is known that recurrent networks of integrate-fire neurons are able to represent distributions given noisy inputs [48]. As a result, all parts of the complete integrate-fire-Snyder estimation algorithm possess the potential for possible biological implementation.

## Discussion

### Bump shape is relatively unimportant, mean latency is critical, integrate-fire-Snyder is a powerful estimation scheme

The above analyses show that as light intensity increases, the dominant noise source transitions from being extrinsic to intrinsic. Furthermore, among intrinsic noise sources, it seems that the performance deterioration caused by QB shape variability is small compared to that introduced by the mean cascade delay. As a result, the relative magnitude of the mean QB arrival time, given a photon, to the mean photon inter-arrival time is a key indicator of noise performance. Such an event timing based description of noise fits well into the Poisson channel framework in which this estimation problem lies.

The relatively small impact of QB shape noise is particularly significant. Previous research using signal to noise ratios found QB amplitude variation responsible for limiting cascade reliability at all frequencies [10]. This diametrically opposite conclusion is likely evidence of why it is important to get the performance metric right [49]. Furthermore, work based on the Poisson-variance approach [9] predicted that at least half of the total noise (extrinsic and intrinsic) was always due to the cascade. This directly belies the extrinsic-intrinsic noise transition described here and elucidates the importance of maintaining a point process approach which properly accounts for signal causality.

The mse_d_ curve exactly describes the optimal distortion given photon noise and only a fixed cascade delay. It therefore provides a distortion lower bound by describing a hypothetical scenario in which all (independent) sources of variability have been removed from the cascade. The ${\text{mse}}_{\text{qb}}^{*}$ curve is derived from the integrate-fire-Snyder algorithm and upper bounds the unknown MMSE given the QB stream. Simulations found that these two bounds were generally in close agreement. This suggests that the integrate-fire-Snyder is likely a good means of converting QBs into estimated photons, over a wide range of intensities. In fact, as shown in Fig 4, the algorithm outperformed several standard machine learning techniques, achieving lower ${\text{mse}}_{\text{qb}}^{*}$ values on the same data. This not only reinforces the integrate-fire-Snyder as a good estimation method but also suggests that, generally, it may be beneficial to reduce a QB stream to an estimated photon stream before performing inference. This seems reasonable as QBs, being responses to individual photons, must have a discrete information structure embedded within their noisy waveforms. Note that, since the integrate-fire-Snyder makes few assumptions on its input and output signals, it may also have applications in more general systems with discrete-sum informational structures.

Work by van Steveninck and Laughlin [50] treated the transduction process as a filtering operation and postulated that one could recover the exact photon times by inverting the filter, subject to fundamental performance limitations. The integrate-fire-Snyder algorithm fits exactly into this framework, albeit in a non-linear point process setting, and thus provides a good answer to this postulation. The algorithm performance suggests that generating a good photon event estimation is sufficient to achieve good ${\text{mse}}_{\text{qb}}^{*}$ and that more complex techniques are unnecessary. However, the use of simplifying techniques that do not fully model process dynamics can also be misleading. There exists a set of (*α*, *k*) for which the noisy integrate-fire-Snyder estimate, ${\text{mse}}_{\text{qb}}^{*}$, outperforms the linear MMSE estimate given the true photon data, ${\text{mse}}_{\text{ph}}^{l}$ (see Fig 6). Any linear estimator using the QB data must do even worse. Performing the analysis under a linear or continuity approximation therefore gives a wrong impression of the noise floor for the system (since the MSE with only photon noise is so high) and will not resolve the more subtle noise source contributions. This results in a spurious picture of phototransduction noise performance. For a more detailed analysis of the dangers of such approximations, when applied to systems which are naturally discrete and causal see Parag and Vinnicombe [51] [30].

For all the stimuli investigated, photon noise was found to be limiting at low light intensities. This is likely due to the photon inter-arrival time being much larger than the delays and widths of QBs. Given this dominance, one can easily convert QBs into estimated photons using naive threshold based methods (see the ‘pure threshold’ curve of Fig 4), as the cascade deterioration has negligible impact on inference. Further, as ${\text{mse}}_{\text{qb}}^{*}\approx {\text{mse}}_{\text{ph}}$ in this region, the true MMSE given QBs can be well approximated with the exact, standard Snyder solution. As intensity increases all MSE curves fall due to the availability of more information. However, cascade based noise becomes visible as the ${\text{mse}}_{\text{qb}}^{*}$ curve significantly separates from the mse_ph_ one. This divergence increases with normalised intensity, *β*. At very high intensities photon noise contributes almost nothing and mse_ph_ → 0 as *β* → ∞.

A major part of this work involved trying to infer the relative contributions of the various intrinsic noise sources. As noted previously, intrinsic noise is composed of dark noise (false positive QBs), QB shape noise (amplitude and width variability), QB latency (mean delay and jitter) and a quantum capture efficiency, QE < 1 (an analogue to false negative QBs). These components are independent of each other and can therefore be treated in isolation. Dark noise is negligible under the conditions of this work and was excluded from analysis. QE is usually high in real invertebrate visual systems and it can be treated as an additional noise component on top of the cascade noise (which involves QB shape and latency). Moreover, simulations found results to be robust to the loss of up to $\frac{1}{3}$ of all input photons (QE = 0.66) (see Supplements S3 Text, S4 and S5 Figs). The integrate-fire-Snyder adapted to lower QEs by simply reducing its trained firing threshold *ζ*, which encodes how much QB charge is believed to represent a photon. As a result, QE can be largely ignored.

The relative noise problem in the medium-high intensity regime was therefore reduced to one of disentangling the relative impact of QB shape and latency. This was initially investigated by comparing the ${\text{mse}}_{\text{qb}}^{*}$ bounds achievable with different noise components excluded from the Nikolic model. If shape noise is removed then the observed QB stream is equivalent to the input photon stream distorted according to the physiological QB latency distribution. This stream was described as empirically delayed. The optimal filter for empirically delayed photons is computationally intractable. However, it was found that applying the standard Snyder filter to the empirically delayed photons gave an MSE upper bound that was in close agreement with the fully noisy ${\text{mse}}_{\text{qb}}^{*}$ (see Supplements S3 Text, S4 and S5 Figs). This hinted that shape noise could be less important than latency. To test this hypothesis it was necessary to construct complementary lower bounds on achievable noise performance.

The QB latency profile can be well described by a normal distribution that matched its mean [52]. Since normal distributions are parameterised solely in terms of their mean and variance, QB latency may then be considered as essentially and independently composed of a mean delay and jitter. Separating the impact of each component therefore makes sense. The mean delay was analysed, because i) unlike jitter it can be examined within an exact inference framework, and ii) fixed delays or dead times have a clear and important impact on causally constrained real time systems such as vision. Additionally, there is some evidence in the literature for dead times and fixed delays being both irreducible and critical to high intensity phototransduction noise performance [52]. This motivated the development of the optimal deterministically delayed Snyder filter, which yielded the mse_d_ curve. This curve provided a lower bound on the remaining error when all cascade variability, including jitter, was removed.

A close correspondence between mse_d_ and ${\text{mse}}_{\text{qb}}^{*}$ was observed across all the light models investigated. This confirmed QB latency as the dominant cascade noise source and suggested that the mean delay in generating a QB from a photon input is critical to cascade noise performance. In fact, when plotted, the most visible difference between ${\hat{x}}_{\text{ph}}\left(t\right)$ and ${\hat{x}}_{\text{qb}}^{*}\left(t\right)$ is a time shift which is of the order of the mean delay. For an illustration of this difference on a more complex light model see Supplement S6 Fig. The cascade therefore essentially encodes discrete events as delayed electrical depolarisations. As a result, by elimination, the actual shape of the latency distribution is not very important and the variable shape of the QB largely insignificant. This conclusion was further investigated by applying the integrate-fire-Snyder to the Nikolic model with all forms of variability turned off. At this setting QBs are of deterministic shape and size, and only the mean delay is acting on the cascade. The resulting MSE curves lay close to and between mse_d_ and ${\text{mse}}_{\text{qb}}^{*}$. This correspondence validated the performance deteriorating dominance of mean delay.

Thus to a first approximation, the complex set of cascade reactions can be replaced with a pure delay on the photon inputs. This conclusion contradicts the work in [9] and reiterates the importance of maintaining a causal, discrete approach. This is especially the case here since pure delays do not affect the common (acausal) mutual information and would largely be neglected in such analyses. However, this work asserts that such delays are important, especially as they may limit the ability of an invertebrate to respond rapidly to stimuli. The dominance of QB delay on cascade noise means that QB shape can be optimised for other performance goals, such as achieving a dynamic range that maximises the input amplitude representation, without affecting cascade accuracy. This shape-latency decoupling allows for more flexible cascade functionality and may underly why experiments have found the QB waveform and latency to be uncorrelated [16].

### Reducing mean delay may not be physiologically possible

Cascade delay appears to be the single largest intrinsic noise component. One may therefore wonder why nature has not further optimised phototransduction to reduce its impact. Reducing delay would not only improve accuracy but also increase visual bandwidth. According to Eckert and Zeil [53] faster phototransduction requires more mitochondria and hence is much more energy intensive. Consequently, improving latency will only be feasible if the organism is willing to devote more energy to visual processes. This explains why flies that must perform more demanding visual tasks, such as Coenosia, have faster cascades than Drosophila [54] [1]. The dominant effect of cascade delay also clarifies why visual light adaptation, which involves the production of faster QBs, is helpful at high intensities. In this regime the latency becomes much more critically limiting and its impact must therefore be, at least partly, countered [14]. A key feature of the adaptation response is a reduced mean delay but a mostly fixed jitter [52]. This seems to support the idea that mean delay is the major source of cascade noise.

Moreover, even if energy constraints were not limiting, significantly improving QB latency and mean delay would still be unlikely. The latency relates to the time taken for a sufficient amount of G proteins and phospholipase C molecules to become activated such that the threshold for opening the TRP channels and generating a normal QB is achieved [15]. A noteworthy reduction in cascade latency would require a reduction in the threshold for opening the TRP channels. However, this would increase the sensitivity of the cascade to spontaneous phospoholipase C and G protein activations which would lead to an increase in dark noise [55]. Thus, mechanistically reducing latency to improve cascade accuracy may simply result in a trade between the limiting noise source and possibly lead to no overall improvement.

### Concluding remarks

This paper has outlined a useful scheme for extracting data from QBs and used this to characterise the main sources of noise in invertebrate phototransduction. By making use of the photon counting nature of the visual system, and point process theory, a consistent and accurate measure of relative noise was achieved that extends the stimuli reconstruction methodology of Bobrowski *et al* [32] and clarifies the until now contradictory results between the work of van-Steveninck and Bialek [10] and those of Lillywhite [3] and Laughlin [9]. Furthermore, this work pinpointed mean delay as the key cascade noise source and emphasised the dangers of using continuity approximations for inherently discrete random systems. Lastly this research fulfils the request of Grewe *et al* [27] for a sensible metric (causal MMSE) which can give a ‘complete description of the system [photoreceptor] performance’.

## Supporting information

S1 Text Snyder point process optimal filtering and optimal linear filtering for the interrupted model. The general non-linear Snyder filter is presented and then solved for the interrupted model. The optimal linear filter for this model is also derived.
(PDF)

S2 Text Multi-state bimodal light models simulate a main light switch with gradual transitions between non-modal states. The bimodal light model is derived and examined in terms of both its similarity to the interrupted model and the extra dynamical complexity it allows.
(PDF)

S3 Text Representative MSE curves for extrinsic and intrinsic noise components on bimodal light models. A 16 state bimodal model is analysed with the integrate-fire-Snyder approach.
(PDF)

S4 Text The Nikolic phototransduction model. Further information (including a mathematical description) about the phototransduction model used in this work is provided.
(PDF)

S1 Fig Markov bimodal light model with 8 states coding for different light intensities. The *a*_*i*_ and *b*_*i*_ are birth and death reaction rates respectively, indicating incremental (nearest neighbour) increases and decreases in light intensity. The *ϵ*_*ij*_ are the modal switches. The rates are chosen to achieve a bimodal state distribution centred on the modal states. The 16 state version of this model was simulated in this work.
(TIF)

S2 Fig Optimal intensity estimate given known photons at t = [0 300] ms for 8 state bimodal model. The more complex Snyder equations for the bimodal model can be decomposed into an exponential solution that is qualitatively similar to that of the interrupted model (and exactly the same when the Markov chain has only 2 states, which are both trivially modal). Observe the discontinuous update at the photon time of 300ms. This model features *ϵ*_36_ = *ϵ*_63_ = *k*.
(TIF)

S3 FigPower spectral density for the 16 state bimodal light intensity.Fast Fourier transforms were used to calculate the frequency response of the 16 state bimodal light model at [*γ*, *ϵ*] = [10, 3*k*]. The extra small amplitude fluctuations (nearest neighbour reactions) about the high amplitude modal switches leads to ‘$\frac{1}{f}$ type’ (precisely *f*^−1.23^) behaviour with *f* indicating frequency. This holds over a reasonably large frequency range. This shows that more naturalistic dynamics that can be achieved using higher state Markov modulated Poisson light models.(TIF)

S4 Fig Photon noise or cascade delay dominates for a bimodal multi-state model. The conclusions of the interrupted model are shown to hold for the more complex bimodal case. All curves converge at low intensity and all curves with additional intrinsic noise converge at higher intensities. Data is for a 16 state model at [*γ*, *ϵ*] = [20, *k*].
(TIF)

S5 Fig Results on dominant noise remain unchanged with *γ* for the multi-state bimodal models. The key conclusions from the filtering analysis across *β* and *γ* remain true for more complex models such as this one. Data is for a 16 state bimodal model at [*γ*, *ϵ*] = [10, 3*k*].
(TIF)

S6 FigEstimated intensities before and after the cascade show it mostly acts as a deterministic delay.The integrate-fire-Snyder conditional estimate ${\hat{x}}_{\text{qb}}^{*}\left(t\right)$ (QE = 0.66, all noise) appears as a delayed version of the Snyder MMSE estimate (only photon noise) ${\hat{x}}_{\text{ph}}\left(t\right)$. The data is for a bimodal 16 state model with [*γ*, *ϵ*] = [20, *k*] and *β* = 100. The normalised relative intensity *β* is set to fall within the parameter regime where intrinsic noise dominates.(TIF)

S7 Fig The Nikolic noise distributions for QBs. The Nikolic model was run over across 9006 QBs and the resulting QB latency, height and area histograms obtained. Observe the mean delay is around 43ms. These distributions are known to match experimental results. In the deterministic Nikolic implementation, all these histograms collapse to their fixed mean values.
(TIF)

S8 Fig The average QB shape from the Nikolic model. The stochastic Nikolic model was run over 9006 QBs and the photocurrent responses averaged. The resulting QB was compared directly with experimental data from Hardie *et al* [13].
(TIF)
