# Supplementary material for: Point process analysis of noise in early invertebrate vision
Source: PLoS Comput Biol. 2017 Oct 27;13(10):e1005687. doi: 10.1371/journal.pcbi.1005687 (PMC5678801; doi:10.1371/journal.pcbi.1005687)
Supplement: S1 Text — The general non-linear Snyder filter is presented and then solved for the interrupted model. The optimal linear filter for this model is also derived. (PDF) [file pcbi.1005687.s001.pdf]

# Point Process Analysis of Noise in Early Invertebrate Vision

Kris V Parag*, Glenn Vinnicombe,
**Control Group, Department of Engineering, University of Cambridge, UK**
**E-mail: kvp23@cam.ac.uk (KVP)**

# Supplementary Information S1

## Snyder Point Process Optimal Filtering

In this work light intensity is described by a hidden Markov process that modulates the rate of a Poisson process. The Poisson process produces photons and the entire model is known as a Markov modulated Poisson process (MMPP). The estimation problem at the front end of the cascade (given only photon noise) is then to reconstruct the Markov light intensity from the resulting photon stream. The Snyder filter is a Bayesian inference technique that provides the optimal or minimum mean squared error (MMSE) solution to this problem [1]. It is the point process analogue of the famous Kalman filter [2]. The Snyder filter is an exact method (no approximations are made on either the state or observation process), that evolves the posterior statistics of the MMPP using differential-difference equations. The solution to these hybrid equations involves a discontinuous change at event (photon) times with a continuous inter-event decaying trajectory. The general construction of the filter follows.

Let $x(t) \in \{0, 1, 2, \ldots, m\} = \mathbb{X}$, be an unobserved, discrete state, continuous time Markov process that represents the fluctuating, dimensionless, normalised light intensity. In this work, the incident light intensity, $\lambda(t)$ is defined by the linear equation $\lambda(t) = \alpha x(t)$ with $\alpha > 0$ ms$^{-1}$. However, the filter description which follows holds for any functional intensity-state description. The intensity modulates an observed Poisson point process $N(t)$ for $0 \leq t \leq s$ (denoted $N_0^s$). Photons are therefore produced from a MMPP with rate $\lambda(t)$. If the causal posterior probability, $\mathrm{P}\left(x(t)|N_0^t\right)$, is described by the $1 \times m$ row vector $q(t)$, then the continuous (differential) component of the Snyder filter is given by equation 1 [1]. The sum $\sum$ is taken across the columns of a vector. Here $R$ is the infinitesimal generator of the Markov process, $\Lambda$ the diagonal rate matrix which controls how the rate of $N(t)$ depends on each $x(t)$ state, and I the identity matrix. $R$ contains all the Markov chain connection rates and sums to zero on each row, while $\Lambda$ has non-negative diagonal values.

$$\frac{dq(t)}{dt} = q(t)\left(R + \left(\sum q(t)\Lambda\right)\mathrm{I} - \Lambda\right) \tag{1}$$

The above set of coupled ordinary differential equations are solved between the event times of $N(t)$ (photon inter-arrival solution). At the $N(t)$ event times the following update is applied with $q(t^-)$ and $q(t^+)$ as the posterior probabilities infitesimally before and after a photon arrival discontinuity at time $t$.

$$q(t^+) = q(t^-)\Lambda\left(\sum q(t^-)\Lambda\right)^{-1} \tag{2}$$

After every photon, the updated posterior from expression 2 is used as a new initial condition for solving equation 1. The above equations work for any $R$ and $\Lambda$. The resulting conditional mean estimator, $\hat{x}(t) = \mathbb{E}\left[x(t)|N_0^t\right]$, which achieves the MMSE: $\mathbb{E}\left[\left(x(t) - \hat{x}(t)\right)^2\right]$ is calculated as: $\hat{x}(t) = \sum_{j \in \mathbb{X}} j\mathrm{P}\left(x(t) = j|N_0^t\right)) = \sum_{j \in \mathbb{X}} jq_j(t)$, with $q_j(t)$ as the $j^{\text{th}}$ element of the posterior vector.

The 2 state interrupted model, used in the main text, substitutes the following matrices into the above equations. The resulting $1 \times 2$ posterior can be written as $q(t) = [q_0 \; q_1]$.

$$R = \begin{bmatrix} -k & k \\ k & -k \end{bmatrix} \qquad \Lambda = \begin{bmatrix} 0 & 0 \\ 0 & \alpha \end{bmatrix}$$

Since each $q_i$ is a probability then $\sum_i q_i = 1$ implies $\sum_i \frac{dq_i}{dt} = 0$. This decouples the vector equation set and reduces it to a single expression in terms of $q_1$ only [1]. Further as $\hat{x}(t) = \sum_{j\in\mathbb{X}} jq_j(t) = q_1$ then the inter-event differential equation directly describes the conditional mean estimate dynamics.

$$\frac{d\hat{x}(t)}{dt} = k - (2k+\alpha)\hat{x}(t) + \alpha\hat{x}(t)^2 \tag{3}$$

Hence, for the interrupted model, the Snyder equations explicitly describe the evolution of the state estimate $\hat{x}$ which is denoted as $\hat{x}_{\text{ph}}$ in the main paper. The solution to the above equation (obtained by completing the square) and its discontinuous update result in equations 1 and 2 of the main text.

## Optimal Linear Filtering for the Interrupted Model

Equation 3 above described the non-linear MMSE inter-event trajectory. This is a quadratic ordinary differential equation in $\hat{x}(t)$ and is derived by maintaining a causal and discrete approach to both the state $x(t)$ and its associated Poisson observations, $N(t)$. If instead a continuous approximation is made on the state dynamics using a linear system, then a new quadratic differential equation in terms of a covariance matrix for the interrupted model, denoted $\Sigma_t$, can be obtained [3]. This allows direct calculation of the linear MMSE $\text{mse}^l_{\text{ph}}$. This is directly comparable to the non-linear MMSE, $\text{mse}_{\text{ph}}$, obtained from the Snyder filter. Both measures are at the front end of the phototransduction cascade (only photon noise).

$$\frac{d\Sigma_t}{dt} = 1 - 4k\Sigma_t - 2\alpha k{\Sigma_t}^2 \implies \text{mse}^l_{\text{ph}}(t) = k\Sigma_t \tag{4}$$

Solving the above equation over $t \geq 0$ by completing the square and integrating (with initial condition, $\Sigma_0 = \frac{1}{4k}$) leads to the explicit solutions below. Here $a = 2\alpha k$, $b = 4k$, $c = 1 + \frac{b^2}{4a}$, $d = \frac{b}{2a}$, $\Sigma_t = \sqrt{\frac{c}{a}}\tanh\left(\sqrt{ca}t + G\right) - d$ and $G = \tanh^{-1}\left(\sqrt{\frac{a}{c}}\left[\frac{1}{b} + d\right]\right)$. The solution is solely a function of the dimensionless parameter $\beta = \frac{\alpha}{k}$, which represents the relative intensity speed.

$$\text{mse}^l_{\text{ph}}(t) = k\sqrt{\frac{c}{a}}\tanh\left(\sqrt{ca}t + G\right) - kd \tag{5}$$

$$\text{mse}^l_{\text{ph}} = \lim_{t\to\infty} \text{mse}^l_{ph}(t) = k\left(\sqrt{\frac{c}{a}} - d\right) = \frac{1}{\beta}\left(\sqrt{1+\frac{\beta}{2}} - 1\right) \tag{6}$$

These equations are only valid for the 2 state interrupted model. However, similar analyses can be performed for any multi-state Markov model by appropriately approximating the chain dynamics so that a dynamical description of the covariance matrix results.

# References


1. Snyder D (1975) Random Point Processes. John Wiley and Sons Inc.

2. Kalman R (1960) A New Approach to Linear Filtering and Prediction Problems. Journal of Basic Engineering 82: 35-45.

3. Snyder D, Miller M (1991) Random Point Processes in Time and Space. Springer-Verlag.

4. Rudemo M (1972) Doubly-Stochastic Poisson Processes and Process Control. Advances in Applied Probability 2: 318-338.

5. Nikolic K, Loizu J, Degenaar P, Toumazou C (2010) A Stochastic Model of the Single Photon Response in Drosophila Photoreceptors. Integrative Biology 2: 354-370.

6. Nikolic K, Loizu J (2013) Drosophila Photo-transduction Simulator. Journal of Open Research Software 1.

7. Hardie R (2012) Phototransduction Mechanisms in Drosophila Microvillar Photoreceptors. WIREs Membr Transp Signal 1: 162-187.

8. Henderson S, Reuss H, R H (2000) Single Photon Responses in Drosophila Photoreceptors and their Regulation by Ca2+. Journal of Physiology 524: 179-194.
