# Supplementary material for: Point process analysis of noise in early invertebrate vision
Source: PLoS Comput Biol. 2017 Oct 27;13(10):e1005687. doi: 10.1371/journal.pcbi.1005687 (PMC5678801; doi:10.1371/journal.pcbi.1005687)
Supplement: S2 Text — The bimodal light model is derived and examined in terms of both its similarity to the interrupted model and the extra dynamical complexity it allows. (PDF) [file pcbi.1005687.s002.pdf]

Point Process Analysis of Noise in Early Invertebrate Vision

Kris V Parag*, Glenn Vinnicombe,
Control Group, Department of Engineering, University of Cambridge, UK
E-mail: kvp23@cam.ac.uk (KVP)

Supplementary Information S2

Multi-state Bimodal Light Models Simulate a Main Light Switch with Gradual Transitions between Non-modal States

As mentioned in the main text, the light models developed here attempt to mimic as many of the properties of non-repeated naturalistic stimuli as possible, while still maintaining analytical tractability. The main paper focussed on a 2 state switching model with uniform state distribution, called the interrupted model. Here this interrupted model is extended to a richer multi-state space of m states, with bimodal Gaussian-like state distributions. These are described by nearest neighbour transitions between all states and a multi-state jump between each modal state of the chain (see **S1 Fig** for an 8 state model). This model represents a complex light source which flickers between two extreme intensities while also possessing small light changes about the extreme modes. The R matrix, which defines the transition rates between states in the model, is obtained by solving for an under-constrained system of equations described by $\pi R = 0$. Here π is a $1 \times m$ vector describing the desired bimodal state distribution.

Let the $m \times m$ generator, R have (i^{th}, j^{th}) element denoted by $R_{i,j}$. Then for the bimodal model, R has diagonals $R_{i,i} = -\Sigma_i$, birth reactions $R_{i,i+1} = a_i$, death reactions $R_{i+1,i} = b_{i+1}$ and modal switch entries $R_{j,l} = \epsilon_{jl}$ and $R_{l,j} = \epsilon_{lj}$. Here i spans the Markov state space, j, l are the modal states and Σ_i is such that each row sum is 0. Setting linear death reactions, $b_{i+1} = ki$ with $k > 0$ and allowing $\epsilon_{jl} = \epsilon_{lj} = \epsilon$ admits the solution for the births as: $a_i = b_{i+1}\frac{\pi_{i+1}}{\pi_i}$ where π_i is the ith component of π. The rate matrix, which describes the relation (linear in this case) between the states and the intensity, is then $\Lambda = \alpha \text{ diag}([0\ 1\ 2\ ...\ m])$ for the m state Markov chain. Note that the k here is the same as that used to define the dimensionless parameters γ and β. While π was chosen in this work to satisfy a bimodal Gaussian description, a rich set of light intensity dynamics are achievable with this model by choosing different stationary distributions and tuning the rates of R.

The power spectral density of the intensity for a bimodal light model at $[m, \gamma, \epsilon] = [16, 10, 3k]$ is given in **S3 Fig**. This demonstrates that the mix of rates and amplitudes from the bimodal Gaussian Markov intensity results in $\frac{1}{f}$ like behaviour over a large intermediate frequency (f) range. This is an example of the more complex behaviour achievable with these models over the 2 state interrupted one. Designing different R matrices can generally encode various types of intensity frequency behaviour. This 16 state model and a similar one at $\gamma = 20$ are examined further in Supplementary Information S3.

While a simple analytical solution to the Snyder filter equations for this model is not available, they can be transformed into a linear equation set in un-normalised probabilities that must then be normalised [4]. These are given below with $q_j(t)$ as the jth component of the normalised posterior vector, $q(t)$ and $r(t)$ is the vector of non-normalised probabilities. Note that $q(0^+)$ indicates an initial condition of an assumed photon at $t = 0$. This is adjusted so that each new photon time serves as a new initial condition. As is standard in Snyder descriptions, the inter-event trajectory resets on every event. The reset or update equation remains as in expression (2) of Supplement S1.

$$r(t) = q(0^+)e^{(R-\Lambda)t} \tag{1}$$

$$q_j(t) = \frac{r_j(t)}{\sum_i r_i(t)} \tag{2}$$

The conditional mean estimate follows as $\hat{x}_{\text{ph}}(t) = \sum_{i=1}^{m} iq_i(t)$. If expression 1 is diagonalised it can be shown that, for any Markov model, the inter-event solutions of $\hat{x}_{\text{ph}}(t)$ involve exponential type decaying

functions. Thus the qualitative nature of the bimodal Snyder solution is the same as the interrupted one, and is shown in **S2 Fig** for the visually simpler 8 state model from **S1 Fig** with $\epsilon = k$. There is always a stable decay, since for a well posed problem, $(R - \Lambda)$ will never have positive eigenvalues. Note that the causal state estimate only involves operations on weighted sums and decaying functions. This is why the filter can be easily described with a neuronal network, as was mentioned in the 'Biological Realisability' section of main text. In summary, the Markov modulated Poisson light descriptions given here allow the encoding of a range of qualitatively different light intensity dynamics without changing the qualitative filtering solution form.

References

1. Snyder D (1975) Random Point Processes. John Wiley and Sons Inc.

2. Kalman R (1960) A New Approach to Linear Filtering and Prediction Problems. Journal of Basic Engineering 82: 35-45.

3. Snyder D, Miller M (1991) Random Point Processes in Time and Space. Springer-Verlag.

4. Rudemo M (1972) Doubly-Stochastic Poisson Processes and Process Control. Advances in Applied Probability 2: 318-338.

5. Nikolic K, Loizu J, Degenaar P, Toumazou C (2010) A Stochastic Model of the Single Photon Response in Drosophila Photoreceptors. Integrative Biology 2: 354-370.

6. Nikolic K, Loizu J (2013) Drosophila Photo-transduction Simulator. Journal of Open Research Software 1.

7. Hardie R (2012) Phototransduction Mechanisms in Drosophila Microvillar Photoreceptors. WIREs Membr Transp Signal 1: 162-187.

8. Henderson S, Reuss H, R H (2000) Single Photon Responses in Drosophila Photoreceptors and their Regulation by Ca2+. Journal of Physiology 524: 179-194.
