# Supplementary material for: Point process analysis of noise in early invertebrate vision
Source: PLoS Comput Biol. 2017 Oct 27;13(10):e1005687. doi: 10.1371/journal.pcbi.1005687 (PMC5678801; doi:10.1371/journal.pcbi.1005687)
Supplement: S3 Text — A 16 state bimodal model is analysed with the integrate-fire-Snyder approach. (PDF) [file pcbi.1005687.s003.pdf]

Point Process Analysis of Noise in Early Invertebrate Vision

Kris V Parag*, Glenn Vinnicombe,
Control Group, Department of Engineering, University of Cambridge, UK
E-mail: kvp23@cam.ac.uk (KVP)

Supplementary Information S3

Representative MSE Curves for Extrinsic and Intrinsic Noise Components on Bimodal Light Models

This section expands and confirms the results of the main text by examining intrinsic and extrinsic noise performance curves for the bimodal model with $m = 16$ states in **S4 Fig and S5 Fig**. All curves have at least extrinsic (photon) noise. Results are not smoothed across repeated runs as in the main text due to computational time (the 16 state model is considerably more complex) and because the relative noise performance must (and does) hold for any given (long enough) light model trajectory (ergodicity). Each point on any curve here is obtained by processing streams with 7000-8000 photons. No curves are presented for the deterministic Nikolic model since it was only used to bolster the results of the main text. Instead, two additional MSE curves are introduced in these figures. These additional curves have also been tested on the interrupted model and results found consistent with those below.

The first is the 'empirical delay' curve which provides a measure of the impact of total QB latency. It is achieved by applying stochastically delayed photons, drawn from the fly phototransduction latency distribution (see **S7 Fig**), to the standard Snyder filter. It upper bounds the contribution of both the mean delay and jitter components of latency noise. The second assesses the contribution of non-unity quantum efficiency (QE) values (the 'all noise, QE = 0.66' curve). This involved randomly thinning an incoming photon stream with Bernoulli probability of 0.66 and then applying it to the Nikolic model to obtain a thinned QB stream. This integrate-fire-Snyder is then applied with training done on the thinned QB stream and original photon stream (no thinning). The resulting MSE curve is an upper bound on all photon and cascade noise. The 'all noise, QE = 1' curve is the standard type of integrate-fire-Snyder result used in the main text and upper bounds all the noise with the exception of QE. The 'deterministic delay' curve, is also as in the main text, and lower bounds the error when all other intrinsic noise, except for the mean delay, is removed. The 'no noise' curves are exact Snyder MMSE values and specify the distortion at the front end of the cascade.

At low β all plots converge, while at higher β the MSE contribution of the intrinsic noise becomes clearly visible. At higher β all the curves which possess intrinsic noise are in close agreement. This suggests mean delay is critically important to cascade noise performance. The similarity of both 'all noise' curves suggest that any QE of at least 0.66 can be well tolerated by the cascade. The closeness of the 'deterministic delay' and 'empirical delay' bounds reinforces the conclusion that QB latency jitter is not as important as the mean delay. At higher γ (lower relative intensity flicker or equivalently lower QB size to switch time) MSE falls because there are more QBs possibly available per switch time. However, the qualitative and relative MSE behaviour with β is identical. Results are therefore consistent across the interrupted and bimodal models. **S6 Fig** illustrates and emphasises the conclusion that mean delay is the key intrinsic noise source. It shows, for the 16 state bimodal model, that $\hat{x}_{\text{qb}}$ (with QE of 0.66) looks like a time lagged version of $\hat{x}_{\text{ph}}$. The apparent delay, which is the most obvious difference between both estimators, is not too far off 50ms. This corresponds well with the true mean cascade delay of 43ms.

References

1. Snyder D (1975) Random Point Processes. John Wiley and Sons Inc.

2. Kalman R (1960) A New Approach to Linear Filtering and Prediction Problems. Journal of Basic Engineering 82: 35-45.

3. Snyder D, Miller M (1991) Random Point Processes in Time and Space. Springer-Verlag.

4. Rudemo M (1972) Doubly-Stochastic Poisson Processes and Process Control. Advances in Applied Probability 2: 318-338.

5. Nikolic K, Loizu J, Degenaar P, Toumazou C (2010) A Stochastic Model of the Single Photon Response in Drosophila Photoreceptors. Integrative Biology 2: 354-370.

6. Nikolic K, Loizu J (2013) Drosophila Photo-transduction Simulator. Journal of Open Research Software 1.

7. Hardie R (2012) Phototransduction Mechanisms in Drosophila Microvillar Photoreceptors. WIREs Membr Transp Signal 1: 162-187.

8. Henderson S, Reuss H, R H (2000) Single Photon Responses in Drosophila Photoreceptors and their Regulation by Ca2+. Journal of Physiology 524: 179-194.
