# Supplementary material for: Point process analysis of noise in early invertebrate vision
Source: PLoS Comput Biol. 2017 Oct 27;13(10):e1005687. doi: 10.1371/journal.pcbi.1005687 (PMC5678801; doi:10.1371/journal.pcbi.1005687)
Supplement: S4 Text — Further information (including a mathematical description) about the phototransduction model used in this work is provided. (PDF) [file pcbi.1005687.s004.pdf]

Point Process Analysis of Noise in Early Invertebrate Vision

Kris V Parag*, Glenn Vinnicombe,
Control Group, Department of Engineering, University of Cambridge, UK
E-mail: kvp23@cam.ac.uk (KVP)

Supplementary Information S4

The Nikolic Phototransduction Model

Nikolic *et al* [5] [6] developed a quantitative Drosophila phototransduction simulator which is herein referred to as the Nikolic model. Coded in Matlab, the software provides a detailed model for the single photon response (QBs) produced by fly photoreceptors. This model splits the Drosophila cascade (component reactions described in the main text) into the key modules of 1) light absorption by Rhodopsin, 2) G protein signal amplification, 3) TRP ion channel kinetics and 4) Ca^{2+} feedback dynamics. The simulator accounts for all known reactions in the cascade and uses approximately 60 parameter values which are either directly found in the literature or estimated by fitting component models to experimental data [6]. It includes the full richness of the phototranduction dynamics and provides mechanisms for dark current noise suppression, feedback and adaptation across a wide range of light intensities [6]. The Nikolic model allows for both deterministic and stochastic models of each module and provides a flexible yet biologically relevant way of investigating not only the noise dynamics of phototransduction, but also that of any G protein based sensory process. [6].

In its deterministic implementation it solves ordinary differential equations of the form on the left of equation 1 with g as the birth rate of some molecule X and r as its death rate. For the stochastic case, the Nikolic model converts the standard rate equations into a Poisson event difference equation that accounts for the production and decay of intrinsically noisy molecular reactions across time. This is done via a Monte-Carlo implementation of the general rate differential equation. This can be seen on the right side of equation 1 below for X. Here Δt is a small time step and $\Pi(\lambda)$ indicates a Poisson distributed variable with mean λ. $\Delta X(t)$ is then known to follow a Skellham type distribution. This equation is appropriately defined for each molecule of each module. More details on these dynamics can be found in [5]. The main text made use of both the deterministic and stochastic Nikolic models.

$$\frac{dX}{dt} = g(X,\, t) - r(X,\, t) \implies \Delta X(t) = \Pi(g(t)\Delta t) - \Pi(r(t)\Delta t) \tag{1}$$

The Nikolic model was adapted for this work because it particularly focuses on getting the noise distributions and statistics correct and allows the flexibility of switching phototransduction noise on/off in a biologically sensible manner [5]. Additionally, the QBs generated by the Nikolic model are known to be consistent with experimental results. This includes, but is not limited to, matching physiological measurements for average QB shape, peak QB current mean and variance, and QB latency distribution [6] [7] [8]. The Nikolic model was used with its default parameter settings in this work, with input photons derived from either the interrupted or bimodal light models. The output of the model was the QB stream defined as Q_0^T in the main text. This consisted of a summed (combined) current time series, that served as the input to the integrate-fire algorithm for photon estimation. Under its stochastic settings (all noise present), the resulting QB noise distributions and the average QB shape, which are of key interest to the main text, are given in **S7 Fig and S8 Fig** respectively. When the Nikolic model was set to its deterministic mode the latency distribution degenerated to simply a point mass with probability 1 at the mean latency value of around 42-43ms and the resulting QBs had identical shapes and sizes.

References

1. Snyder D (1975) Random Point Processes. John Wiley and Sons Inc.

2. Kalman R (1960) A New Approach to Linear Filtering and Prediction Problems. Journal of Basic Engineering 82: 35-45.

3. Snyder D, Miller M (1991) Random Point Processes in Time and Space. Springer-Verlag.

4. Rudemo M (1972) Doubly-Stochastic Poisson Processes and Process Control. Advances in Applied Probability 2: 318-338.

5. Nikolic K, Loizu J, Degenaar P, Toumazou C (2010) A Stochastic Model of the Single Photon Response in Drosophila Photoreceptors. Integrative Biology 2: 354-370.

6. Nikolic K, Loizu J (2013) Drosophila Photo-transduction Simulator. Journal of Open Research Software 1.

7. Hardie R (2012) Phototransduction Mechanisms in Drosophila Microvillar Photoreceptors. WIREs Membr Transp Signal 1: 162-187.

8. Henderson S, Reuss H, R H (2000) Single Photon Responses in Drosophila Photoreceptors and their Regulation by Ca2+. Journal of Physiology 524: 179-194.
