# Supplementary figures and images for: Point process analysis of noise in early invertebrate vision
Source: PLoS Comput Biol. 2017 Oct 27;13(10):e1005687. doi: 10.1371/journal.pcbi.1005687 (PMC5678801; doi:10.1371/journal.pcbi.1005687)

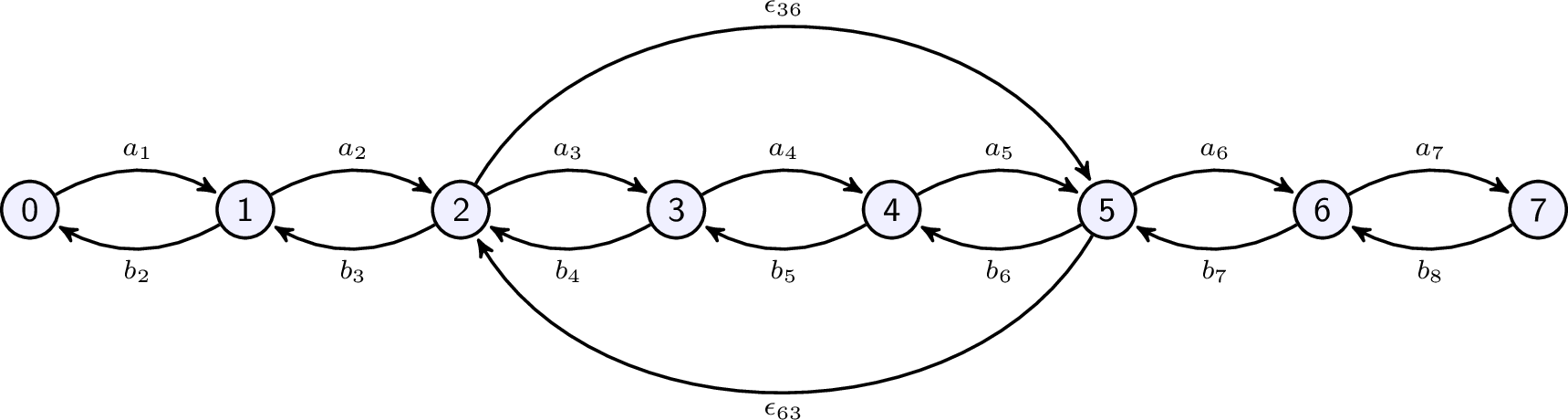

Supplement: S1 Fig — The ai and bi are birth and death reaction rates respectively, indicating incremental (nearest neighbour) increases and decreases in light intensity. The ϵij are the modal switches. The rates are chosen to achieve a bimodal state distribution centred on the modal states. The 16 state version of this model was simulated in this work. (TIF) [file pcbi.1005687.s005.tif]

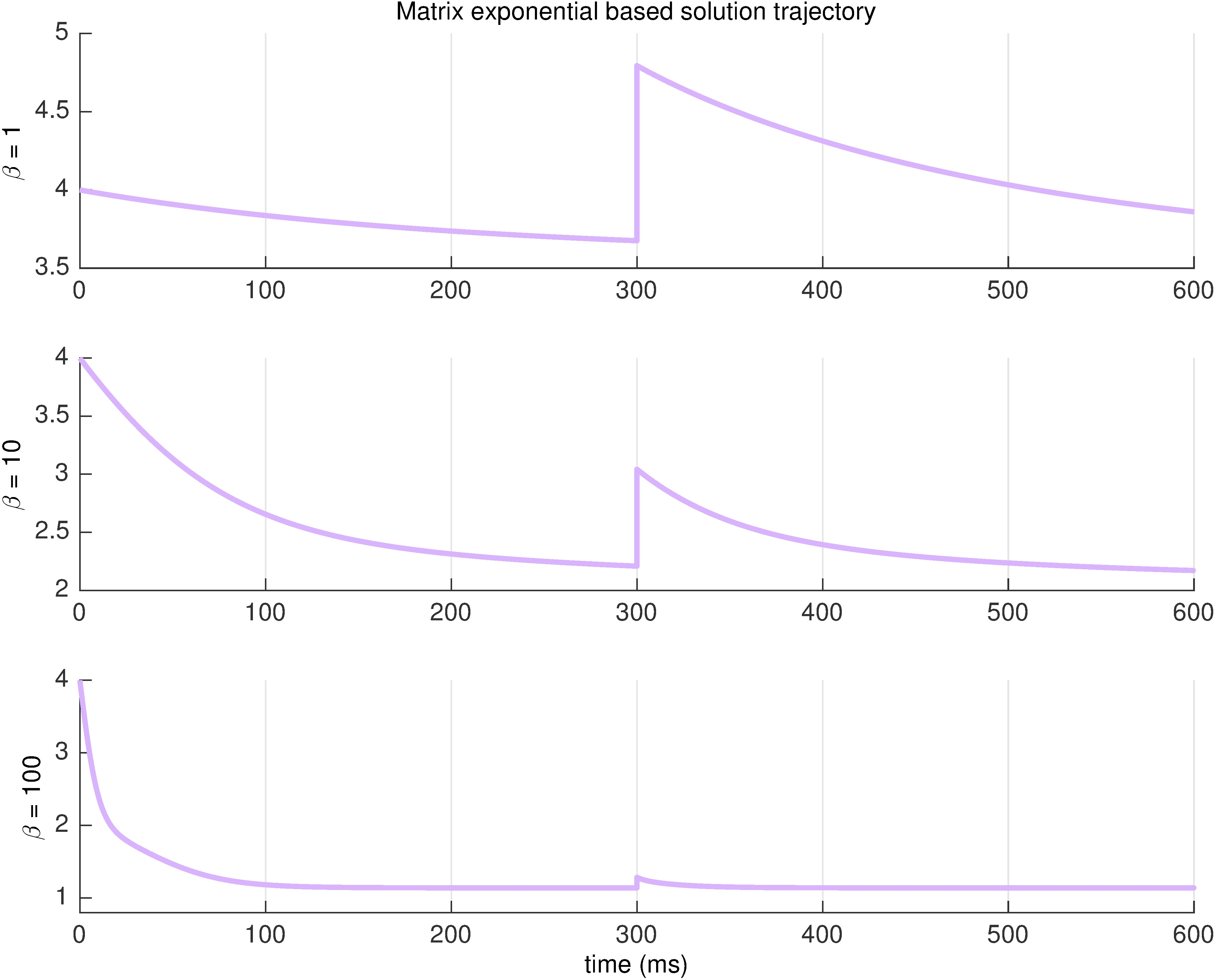

Supplement: S2 Fig — The more complex Snyder equations for the bimodal model can be decomposed into an exponential solution that is qualitatively similar to that of the interrupted model (and exactly the same when the Markov chain has only 2 states, which are both trivially modal). Observe the discontinuous update at the photon time of 300ms. This model features ϵ36 = ϵ63 = k. (TIF) [file pcbi.1005687.s006.tif]

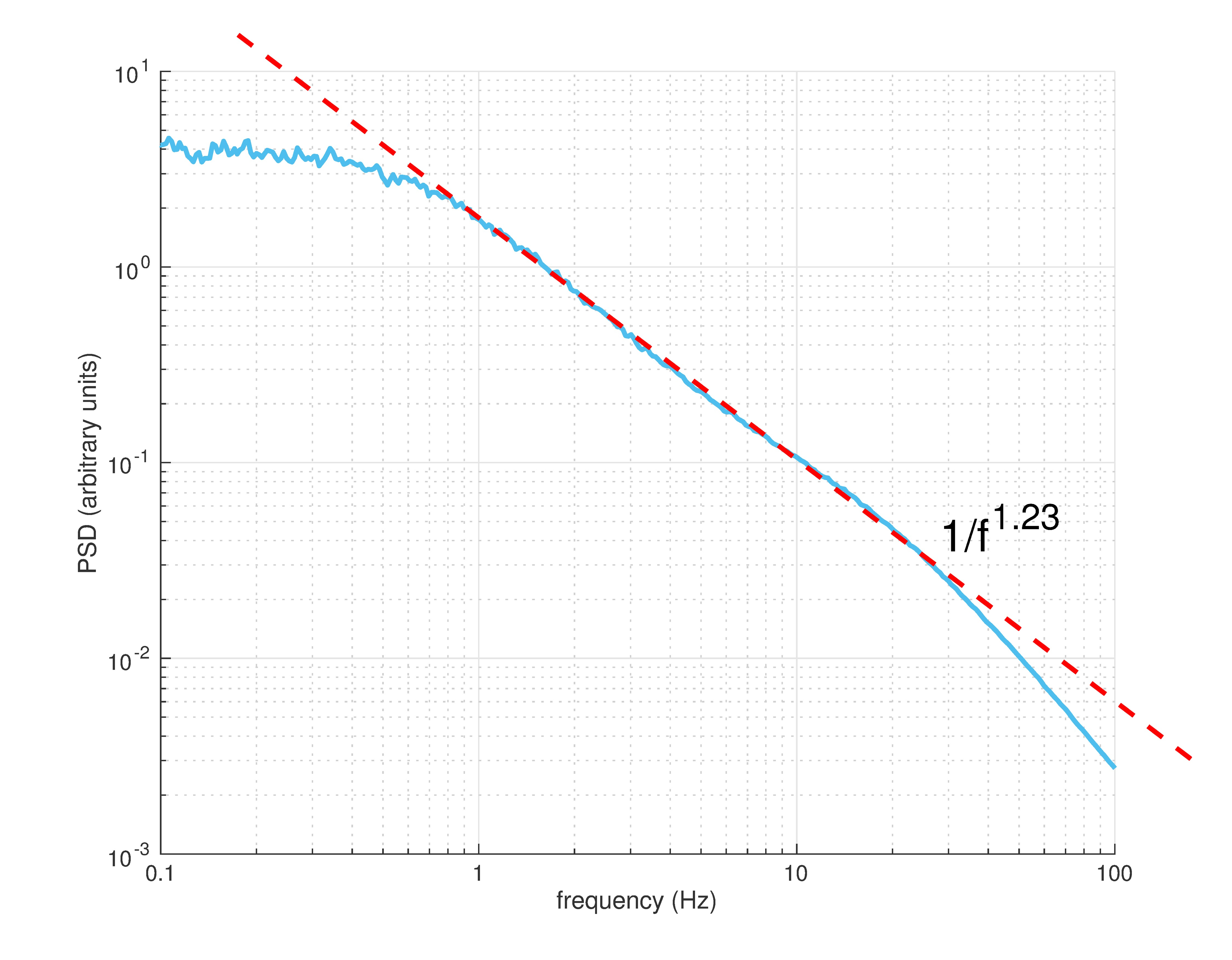

Supplement: S3 Fig — Fast Fourier transforms were used to calculate the frequency response of the 16 state bimodal light model at [γ, ϵ] = [10, 3k]. The extra small amplitude fluctuations (nearest neighbour reactions) about the high amplitude modal switches leads to ‘1f type’ (precisely f−1.23) behaviour with f indicating frequency. This holds over a reasonably large frequency range. This shows that more naturalistic dynamics that can be achieved using higher state Markov modulated Poisson light models. (TIF) [file pcbi.1005687.s007.tif]

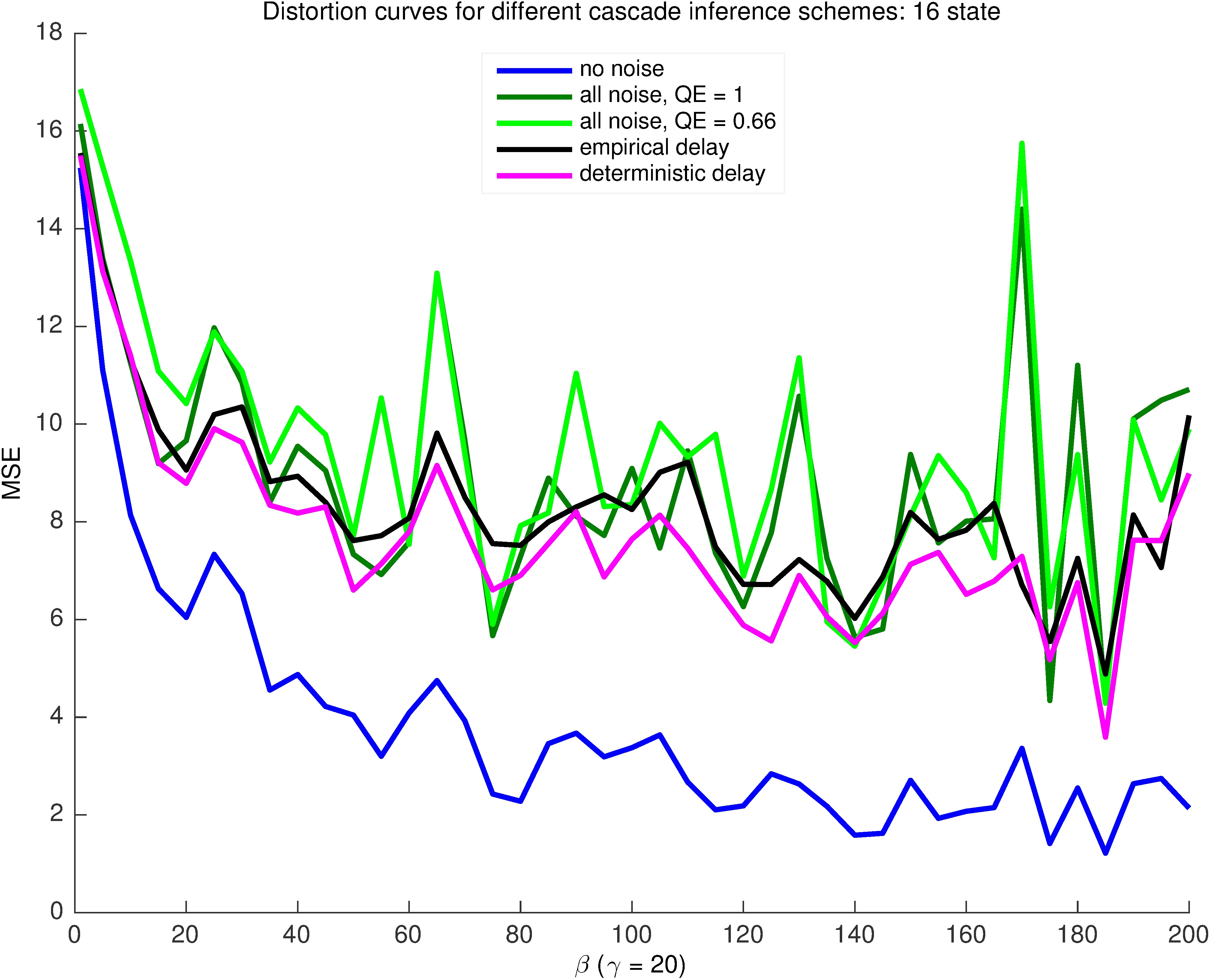

Supplement: S4 Fig — The conclusions of the interrupted model are shown to hold for the more complex bimodal case. All curves converge at low intensity and all curves with additional intrinsic noise converge at higher intensities. Data is for a 16 state model at [γ, ϵ] = [20, k]. (TIF) [file pcbi.1005687.s008.tif]

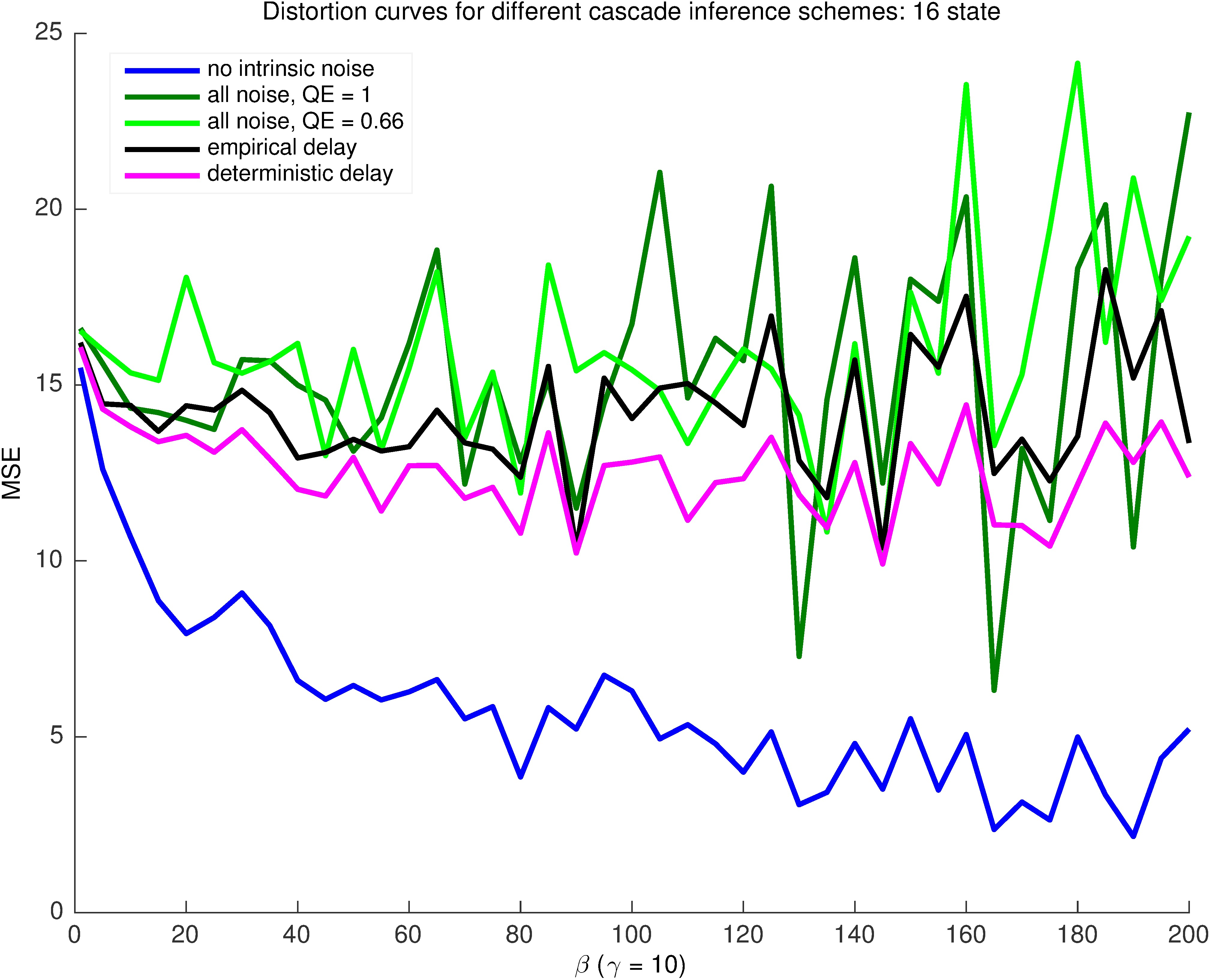

Supplement: S5 Fig — The key conclusions from the filtering analysis across β and γ remain true for more complex models such as this one. Data is for a 16 state bimodal model at [γ, ϵ] = [10, 3k]. (TIF) [file pcbi.1005687.s009.tif]

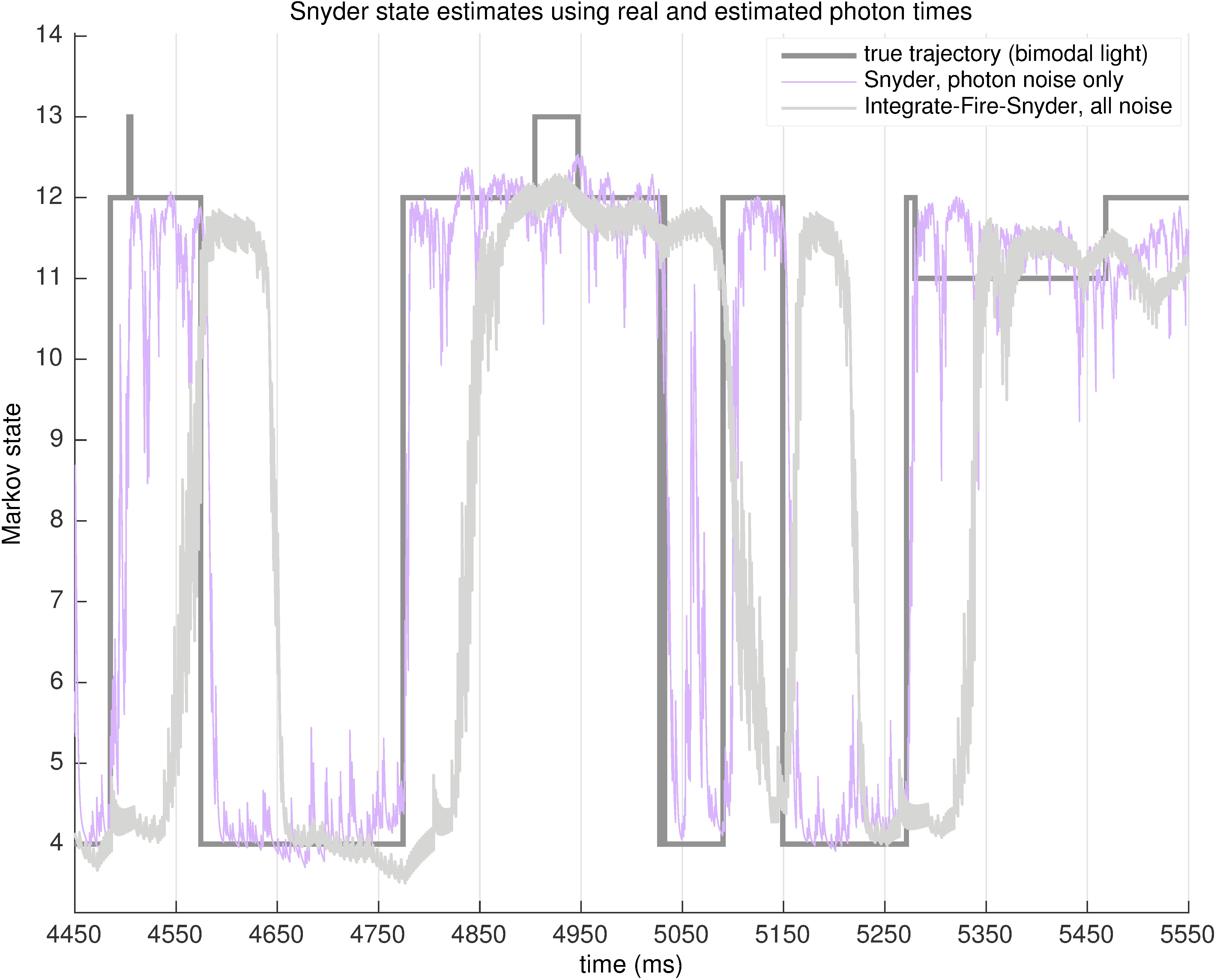

Supplement: S6 Fig — The integrate-fire-Snyder conditional estimate x^qb*(t) (QE = 0.66, all noise) appears as a delayed version of the Snyder MMSE estimate (only photon noise) x^ph(t). The data is for a bimodal 16 state model with [γ, ϵ] = [20, k] and β = 100. The normalised relative intensity β is set to fall within the parameter regime where intrinsic noise dominates. (TIF) [file pcbi.1005687.s010.tif]

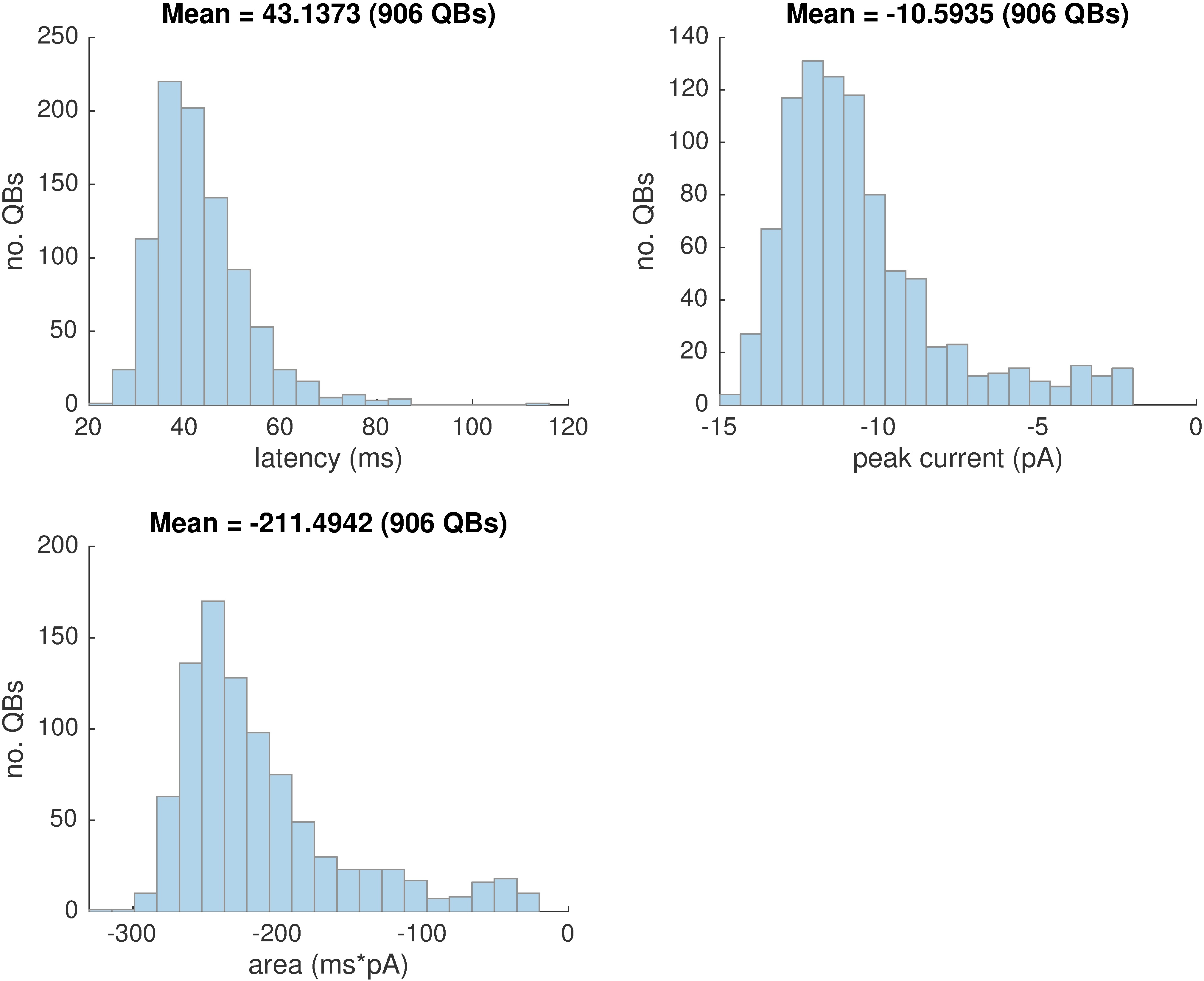

Supplement: S7 Fig — The Nikolic model was run over across 9006 QBs and the resulting QB latency, height and area histograms obtained. Observe the mean delay is around 43ms. These distributions are known to match experimental results. In the deterministic Nikolic implementation, all these histograms collapse to their fixed mean values. (TIF) [file pcbi.1005687.s011.tif]

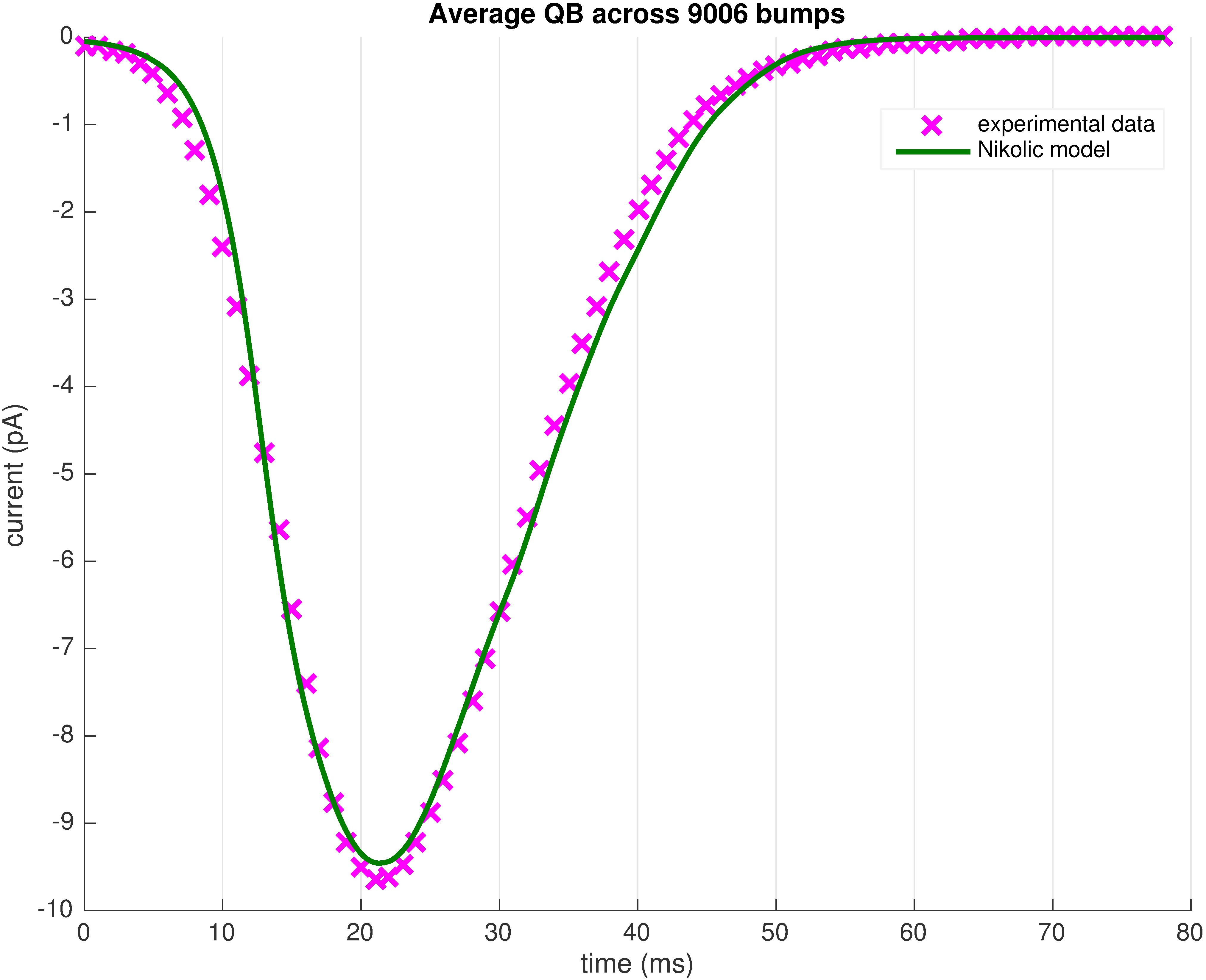

Supplement: S8 Fig — The stochastic Nikolic model was run over 9006 QBs and the photocurrent responses averaged. The resulting QB was compared directly with experimental data from Hardie et al [13]. (TIF) [file pcbi.1005687.s012.tif]
